# Concise Syntheses of Marine (Bis)indole Alkaloids Meridianin C, D, F, and G and Scalaridine A via One-Pot Masuda Borylation-Suzuki Coupling Sequence

**DOI:** 10.3390/molecules27072233

**Published:** 2022-03-30

**Authors:** Marco Kruppa, Gereon A. Sommer, Thomas J. J. Müller

**Affiliations:** Institut für Organische Chemie und Makromolekulare Chemie, Heinrich-Heine-Universität Düsseldorf, Universitätsstrasse 1, D-40225 Düsseldorf, Germany; marco.kruppa@hhu.de (M.K.); gereon.sommer@hhu.de (G.A.S.)

**Keywords:** natural products, one-pot reactions, alkaloids, Masuda borylation–Suzuki coupling

## Abstract

*N*-Protected 3-iodoindoles were reacted with (di)azine halides in a sequentially Pd-catalyzed one-pot fashion, i.e., by Masuda borylation–Suzuki coupling (MBSC) sequence. This methodology was successfully applied to the concise syntheses of marine indole alkaloids meridianin C, D, F, and G, as well as to the bisindole alkaloid scalaridine A, which were obtained in moderate to excellent yield.

## 1. Introduction

**Figure 1:**
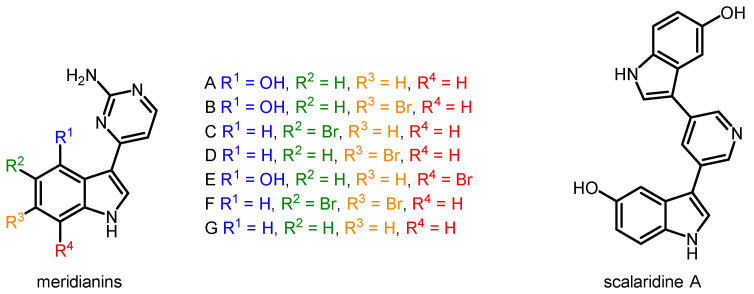
Indole alkaloids meridianins A–G from tunicate *Aplidium meridianum* and scalaridine A from marine sponge *Scalarispongia* sp.

Marine flora and fauna display an extraordinary chemical diversity and harbor an enormous richness of natural products [1]. Every year, a large number of new natural products are discovered and studied, with nearly 1500 new compounds published in 2019 alone [2]. Since natural products often exhibit biological activity, the isolation and characterization of novel marine metabolites play major roles in the development of active ingredients and agrochemicals [3]. Indole alkaloids have been isolated from various marine sources, such as sponges, tunicates, red alga, acorn worms, and symbiotic bacteria [4], and are part of numerous in-depth studies due to their frequent bioactivities, such as cytotoxicity [5], antiviral [6], antimicrobial [7], antifungal [8], anti-inflammatory [9], and antiserotonin [10] properties. Interesting representatives amongst indole alkaloids include meridianins A–G, isolated from the tunicate *Aplidium meridianum* [11,12], described as potent inhibitors of various protein kinases [13], or the bisindole scalaridine A, discovered in the sponge *Scalarispongia* sp., exhibiting cytotoxicity against human leukemia cells (Figure 1) [14].

The concatenation of a Masuda borylation [15,16] and a Suzuki coupling has been described in literature [17,18,19,20,21]; however, we conceptualized a general protocol for coupling of a broad spectrum of heterocycles [22,23,24], in particular a broad selection of 7-azaindoles, such as meriolins [25,26,27,28,29,30,31,32], which are potent kinase inhibitors [33,34,35,36], and apoptosis inducers were readily accessed [37]. Most characteristically, the Masuda borylation–Suzuki coupling (MBSC) sequence takes advantage of the concept of a single catalyst system without the addition of another catalyst loading in the sense of a sequentially Pd-catalyzed one-pot process [38,39]. In the first Pd-catalyzed step the (hetero)aryl halide is borylated with pinacolborane in the presence of triethylamine to scavenge the hydrohalide, furnishing the (hetero)aryl pinacolboronate. This borylated derivative is further reacted with a second (hetero)aryl halide in the sense of a Suzuki reaction in the same reaction vessel to provide an unsymmetrically substituted bis(hetero)aryl product.

**Figure 2:**
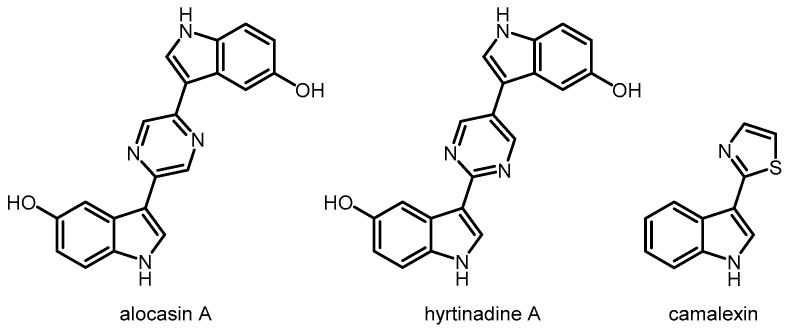
Natural products alocasin A, hyrtinadine A and camalexin synthesized via MBSC sequence.

As illustrations of this powerful MBSC protocol, several indole-containing natural products, such as meridianins A and G (Figure 1) [22], the bisindoles hyrtinadine A [23], alocasin A, and their analogues as efficient MRSA (methicillin-resistant *Staphylococcus aureus*) antibacterials [40], as well as the thiazole containing alkaloid camalexin [24] were synthesized in a concise fashion (Figure 2).

The first synthesis of meridianins set out from transforming 3-acetylindoles into the corresponding enaminones and subsequent cyclization with guanidine [25,26,28]. Later, cycloaddition of nitrosoarenes with alkynylpyrimidines [29], and Pd-catalyzed Cacchi ring-building indole synthesis [31] were introduced as shorter synthetic routes. Employing a three-component carbonylative alkynylation as a key step, followed by cycloaddition, we disclosed a concise two-step protocol [41]. Already in 2000, Suzuki coupling was recognized as a key step towards syntheses of selected meridianins [32], and in 2011 we showed that the MBSC is well suited for the synthesis of meridianins A and G [22]. 

Total syntheses of scalaridine A have been achieved using Ir-catalyzed C-H borylation and Pd-catalyzed Suzuki coupling as key steps [42], as well as several Pd-catalyzed steps including hydrostannylation and Kosugi–Migita–Stille cross-coupling in a multistep synthesis [43], and a Cu-catalyzed Suzuki–Miyaura approach starting from indole boronates in a two-step fashion [44].

Herein, we report the syntheses of the bromo substituted meridianins C, D, and F, as well as meridianin G, and the synthesis of scalaridine A via one-pot Masuda borylation–Suzuki coupling sequence in mostly good yields.

## 2. Results and Discussion

**Scheme 1:**
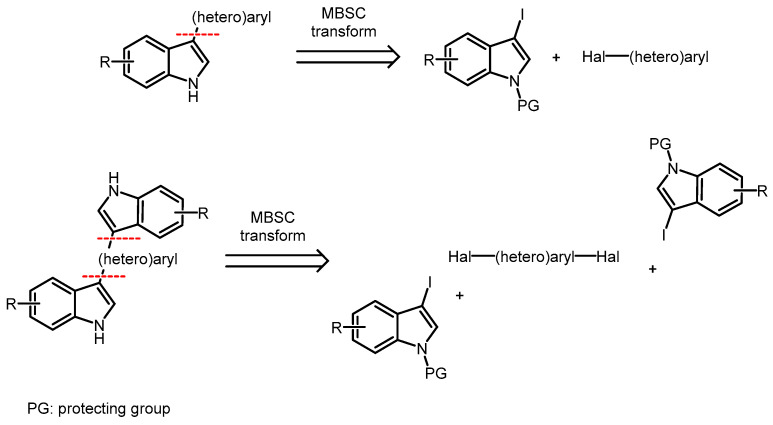
General MBSC transform of (hetero)aryl indoles and its extension to bridged bisindoles.

The MBSC sequence represents an elegant transform for the retrosynthetic analysis of complex heteroaromatic biaryl systems from (hetero)aryl halides as starting materials in a sequentially Pd-catalyzed one-pot procedure (Scheme 1).

**Scheme 2:**
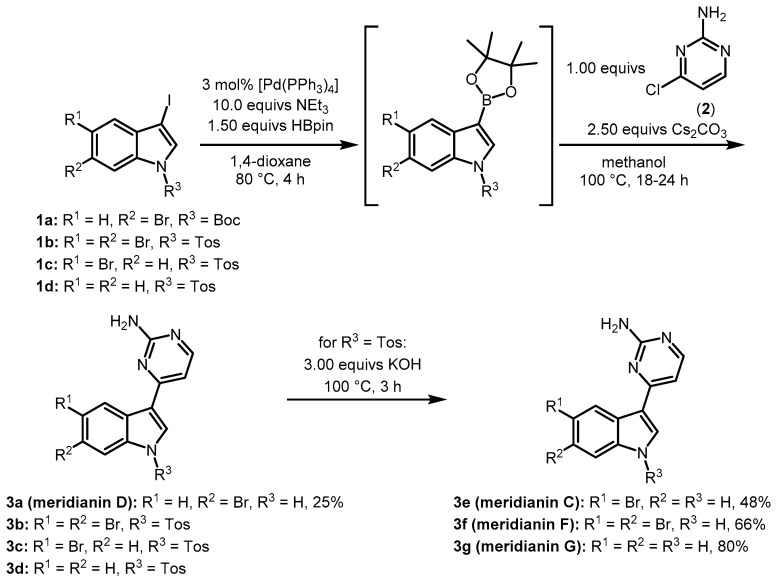
Synthesis of meridianins C, D, F, and G by MBSC sequence.

Therefore, in analogy to the synthesis of meridianins A and G [22], our approach to meridianins C, D, F, and G starts from *N*-Boc- and *N*-tosyl-protected 3-iodoindoles **1a**–**d**. The starting material is prepared in a one-pot process of selective iodation in position three and subsequent *N*-protection according to Witsulski’s [45] and our protocols [23,24]. The indoles **1** are converted to the boronate intermediates by Masuda borylation in the presence of tetrakis(triphenylphosphane)palladium(0), pinacolyl borane (HBpin), and triethylamine in dry 1,4-dioxane (Scheme 2).

After full conversion of the indole **1**, methanol is added to scavenge the remaining excess of HBpin. Subsequent addition of cesium carbonate and 4-chloropyrimidine-2-amine (**2**) initiates the Suzuki coupling reaction in the same reaction vessel. The Boc protecting group is cleaved under the Suzuki conditions furnishing the natural product meridianin D (**3a**) in a yield of 25%. Although the concomitant cleavage of the protecting group during the Suzuki step is elegant, it turned out that this does not work equally well in all substrate combinations [24,37,40]. Indeed, the tosyl group is more robust and can be equally well cleaved upon reaction of the tosylated intermediates **3b**–**d** in the presence of potassium hydroxide in an additional step in the one-pot process to give access to meridianin C (**3e**) (48%), meridianin F (**3f**) (66%), and meridianin G (**3g**) (80%). The analytic data are in excellent agreement with the published data of the isolated natural compounds [11,12] (for the comparison of the NMR data, see Supplementary Materials, Chapter S3). Moreover, *N*-tosyl protected indoles **1** appear to be more stable and more practical than *N*-Boc protected substrates, because no dehalogenation is observed by storage at room temperature.

**Scheme 3:**
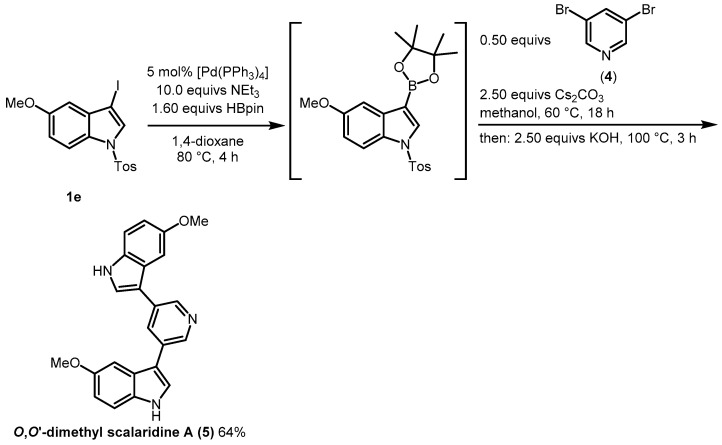
Synthesis of the natural product precursor *O*,*O*′-dimethyl scalaridine A (**5**).

For the synthesis of scalaridine A (**7**), 3-iodo-5-methoxy-1-tosyl-1*H*-indole (**1e**) is employed as a starting material and similarly converted to the respective pinacolyl boronate intermediate in the sense of a Masuda borylation (Scheme 3). After full conversion, methanol is added to scavenge the excess of HBpin. In comparison to the meridianin protocol, increasing the amount of catalyst and HBpin is favorable considering two Masuda borylations per *N*-heterocyclic-bridged bisindole. Then, cesium carbonate and half an equivalent of 3,5-dibromopyridine (**4**) are added for the concluding pseudo three-component Suzuki coupling. Addition of potassium hydroxide cleaves the tosyl group and the alkaloid precursor *O*,*O*′-dimethyl scalaridine A (**5**) is obtained in a yield of 64%.

**Scheme 4:**
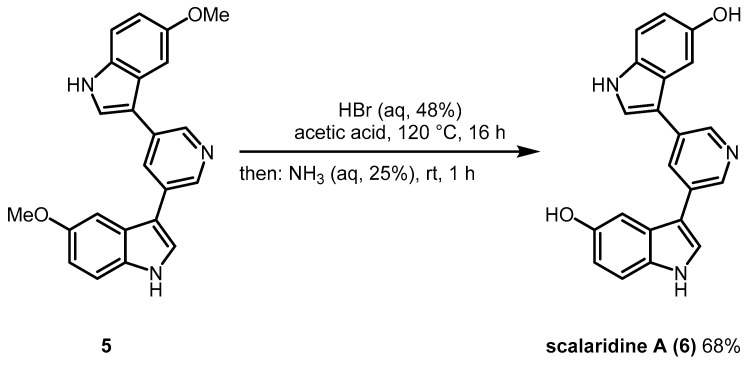
Synthesis of scalaridine A (**6**) by demethylation of *O*,*O*′-dimethyl scalaridine A.

The total synthesis is completed by twofold demethylation [46] of **5** in refluxing acetic acid with hydrobromic acid furnishing the bisindole alkaloid scalaridine A (**6**) in a good yield (Scheme 4). The disappearance of the signal for the methyl groups (*δ* 3.82) and appearance of the OH signal (*δ* 8.81) in the ^1^H NMR spectrum indicates the formation of the natural compound **6** (see experimental section). The overall yield over 2 steps is 44% (starting from **1e**).

## 3. Materials and Methods

### 3.1. General Considerations

All cross-coupling reactions were carried out in oven-dried Schlenk tubes under nitrogen atmosphere. By using MBraun system MB-SPS-800*,* dry 1,4-dioxane was obtained. Dry triethylamine was stored in a Schlenk flask with potassium hydroxide pallets under nitrogen atmosphere. The used *N*-protected 3-iodo-1*H*-indoles **1** were prepared using a literature-known one-pot process [23,24,45]. 4-chloropyrimidine-2-amine (**2**) was synthesized by suspending 2,4-dichloroprimidine in ammonia (aq, 5%) for 2 d [47]. All other used chemicals were purchased at Sigma-Aldrich Chemie GmbH (St. Louis, MO, USA), Acros Organics (Waltham, MA, USA), ABCR GmbH & CO. KG (Karlsruhe, Germany), Alfa Aesar GmbH (Tewksbury, MA, USA) and Merck Serono KGaA (Darmstadt, Germany) and used as supplied.

For purification of the reaction mixtures, a flash chromatography was performed on silica gel 60 (0.015–0.040 mm) from Macherey-Nagel GmbH & Co. KG under a pressure of 2 bar. Therefore, the crude reaction mixtures were adsorbed on Celite^®^ 545 (0.02–0.10 mm) from Macherey-Nagel GmbH & Co. KG. For TLC Silica gel 60 F254 6 × 6 cm^2^ aluminum sheets by Macherey-Nagel GmbH & Co. KG were used. The spots were detected with UV light at 254 and 365 nm. ^1^H, ^13^C, and 135-DEPT NMR spectra were recorded on *Bruker Avance III 300* and *Bruker* *Acance III 600* spectrometers. DMSO-d6 was used as deuterated solvents. For ^1^H spectra, the residual proton signal of the deuterated solvent was locked as the internal standard (DMSO-d_6_, *δ* H 2.50, *δ* C 39.5). The multiplicities of signals were abbreviated as follows: s: singulet, d: doublet, t: triplet, and dd: doublet of doublets. The types of carbon atoms were abbreviated as follows: CH_3_: primary carbon atom, CH_2_: secondary carbon atom, CH: tertiary carbon atom, and C_quat_: quartary carbon atom. For determination a 135-DEPT NMR spectra was used. Mass spectra were measured on Varian MAT 311 A. All peaks with an intensity of >10% corresponding to the base peak were stated. The melting points (uncorrected) were measured on Reichert-Jung S3 Thermovar [48]. Elementary analysis was carried out in the micro analytical laboratory of Institut für Pharmazeutische und Medizinische Chemie der Heinrich-Heine-Universität, Düsseldorf.

### 3.2. Synthesis of Meridianin D (***3a***)

[Pd(PPh_3_)_4_] (19 mg, 19 μmol, 3 mol %) and the *tert*-butyl 6-bromo-3-iodo-1*H*-indole-1-carboxylate (**1a**) (278 mg, 0.66 mmol) were placed in a dry screw-cap Schlenk vessel with a septum and a magnetic stir bar under nitrogen atmosphere and suspended in dry 1,4-dioxane (5.00 mL). After the addition of dry triethylamine (0.92 mL, 6.59 mmol) and 4,4,5,5-tetramethyl-1,3,2-dioxaborolane (0.14 mL, 0.99 mmol) the mixture was stirred at 80 °C (preheated oil bath) for 4 h (monitored by TLC). After cooling to room temperature, methanol (5.00 mL), cesium carbonate (0.54 g, 1.65 mmol), and 4-chloropyrimidine-2-amine (**2**) [47] (85 mg, 0.66 mmol) were added. Then, the mixture was stirred at 100 °C (preheated oil bath) for 18 h. After cooling to room temperature, the solution was diluted with dichloromethane and adsorbed onto Celite^®^. The solvent was removed under reduced pressure and the product was purified by flash chromatography on silica gel (eluent: dichloromethane/methanol/ammonia (aqueous 25%) 100:5:1). The obtained solid was dried under vacuo at 60 °C for 3 to 4 days. Compound **3a** (43 mg, 25%) was obtained as a yellow solid, Mp 211–213 °C (217–221 °C [26]). R_f_ (dichloromethane/methanol/ammonia (aqueous 25%) 100:5:1): 0.32.

^1^H NMR (300 MHz, DMSO-d_6_): *δ* 6.46 (s, 2H), 7.00 (d, *J* = 5.3 Hz, 1H), 7.23 (dd, *J* = 8.5, 1.9 Hz, 1H), 7.62 (d, *J* = 1.8 Hz, 1H), 8.11 (d, *J* = 5.3 Hz, 1H), 8.23 (d, *J* = 2.9 Hz, 1H), 8.56 (d, *J* = 8.5 Hz, 1H), 11.78 (s, 1H). ^13^C NMR (75 MHz, DMSO-d_6_): *δ* 105.2 (CH), 113.2 (C_quat_), 113.3 (C_quat_), 113.7 (CH), 124.5 (CH), 124.6 (CH), 127.0 (C_quat_), 129.6 (CH), 135.7 (C_quat_), 157.1 (CH), 162.2 (C_quat_), 163.5 (C_quat_). HR-MS (ESI) (*m*/*z*) calcd for (C_12_H_9_^79^BrN_4_ + H)^+^: 289.0088; Found: 289.0081.

### 3.3. Synthesis of Meridianin C (***3e***), Meridianin F (***3f***), and Meridianin G (***3g***)

#### 3.3.1. General Procedure for the Synthesis of Meridianins **3e**–**g** via MBSC Sequence

[Pd(PPh_3_)_4_] (19 mg, 19 μmol, 3 mol %) and the 3-iodo-1-tosyl-1*H*-indole **1** (0.66 mmol) were placed in a dry screw-cap Schlenk vessel with a septum and a magnetic stir bar under nitrogen atmosphere and were suspended in dry 1,4-dioxane (5.00 mL). After the addition of dry triethylamine (0.92 mL, 6.59 mmol) and 4,4,5,5-tetramethyl-1,3,2-dioxaborolane (0.14 mL, 0.99 mmol) the mixture was stirred at 80 °C (preheated oil bath) for 4 h (monitored by TLC). After cooling to room temperature methanol (5.00 mL), cesium carbonate (0.54 g, 1.65 mmol) and 4-chloropyrimidine-2-amin (**2**) [47] (85 mg, 0.66 mmol) were added. Then, the mixture was stirred at 100 °C (preheated oil bath) for 15 h. The solution was again cooled to room temperature before potassium hydroxide (0.11 g, 1.98 mmol) was added. After the mixture was allowed to cool down to room temperature it was diluted with dichloromethane and adsorbed onto Celite^®^. The solvent was removed under reduced pressure and the product was purified by flash chromatography on silica gel (eluent: dichloromethane/methanol/ammonia (aqueous 25%) (100:5:1)). The obtained solid was dried in vacuo at 60 °C for 4 d.

#### 3.3.2. Synthesis of 4-(5-Bromo-1H-indol-3-yl)pyrimidin-2-amine, Meridianin C (**3e**)

According to the general procedure starting from 5-bromo-3-iodo-1-tosyl-1*H*-indole (**1c**) compound **3e** (91 mg, 48%) was isolated as a yellow solid, Mp 208–210 °C (238–240 °C [41]). R_f_ (dichloromethane/methanol/ammonia (aqueous 25%) 100:5:1): 0.25.

^1^H NMR (300 MHz, DMSO-d_6_): *δ* 6.50 (s, 2H), 7.01 (d, *J* = 5.4 Hz, 1H), 7.29 (dd, *J* = 8.6, 2.0 Hz, 1H), 7.41 (d, *J* = 8.6 Hz, 1H), 8.11 (d, *J* = 5.3 Hz, 1H), 8.26 (d, *J* = 2.9 Hz, 1H), 8.76 (d, *J* = 2.0 Hz, 1H), 11.86 (s, 1H). ^13^C NMR (75 MHz, DMSO-d_6_): *δ* 105.2 (CH), 113.2 (C_quat_), 113.3 (C_quat_), 113.7 (CH), 124.5 (CH), 124.6 (CH), 127.0 (C_quat_), 129.6 (CH), 135.7 (C_quat_), 157.0 (CH), 162.2 (C_quat_), 163.5 (C_quat_). HR-MS (ESI) (*m*/*z*) calcd for (C_12_H_9_^79^BrN_4_ + H)^+^: 289.0088; Found: 289.0083. Anal calcd for C_12_H_9_BrN_4_ (289.1): C 49.85, H 3.14, N 19.38; Found: C 50.04, H 3.14, N 19.09.

#### 3.3.3. Synthesis of 4-(5,6-Dibromo-1H-indol-3-yl)pyrimidin-2-amine, Meridianin F (**3f**)

According to the general procedure starting from 5,6-dibromo-3-iodo-1-tosyl-1*H*-indole (**1b**) compound **3f** (0.160 g, 66%) was obtained as a colorless solid Mp 290–292 °C (167–169 °C [28]). R_f_ (dichloromethane/methanol/ammonia (aq, 25%) 100:5:1): 0.22.

^1^H NMR (300 MHz, DMSO-d_6_): *δ* 6.56 (s, 2H), 7.02 (d, *J* = 5.3 Hz, 1H), 7.85 (s, 1H), 8.14 (d, *J* = 5.3 Hz, 1H), 8.31 (d, *J* = 1.7 Hz, 1H), 8.97 (s, 1H), 11.94 (s, 1H). ^13^C NMR (75 MHz, DMSO-d_6_): *δ* 105.1 (CH), 113.2 (C_quat_), 114.9 (C_quat_), 116.1 (C_quat_), 116.5 (CH), 126.1 (C_quat_), 126.5 (CH), 130.4 (CH), 136.7 (C_quat_), 157.3 (CH), 161.8 (C_quat_), 163.5 (C_quat_). HR-MS (ESI) (*m*/*z*) calcd for (C_12_H_8_^79^Br_2_N_4_ + H)^+^: 366.9188; Found: 366.9171. Anal calcd for C_12_H_8_Br_2_N_4_ (368.03): C 39.16, H 2.19, N 15.22; Found: C 39.36, H 2.36, N 14.93.

#### 3.3.4. Synthesis of 4-(1H-indol-3-yl)pyrimidin-2-amine, Meridianin G (**3g**)

According to the general procedure starting from 3-iodo-1-tosyl-1*H*-indole (**1d**) compound **3g** (111 mg, 80%) was isolated as a colorless solid, Mp 190–191 °C (195–197 °C [22]). R_f_ (dichloromethane/methanol/ammonia (aqueous 25%) 100:5:1): 0.26.

^1^H NMR (600 MHz, DMSO-d_6_): *δ* 6.41 (s, 2H), 7.01 (d, *J* = 5.3 Hz, 1H), 7.12 (td, *J* = 7.4, 6.9, 1.1 Hz, 1H), 7.17 (ddd, *J* = 8.1, 6.9, 1.3 Hz, 1H), 7.44 (d, *J* = 8.0 Hz, 1H), 8.10 (d, *J* = 5.3 Hz, 1H), 8.19 (d, *J* = 2.9 Hz, 1H), 8.58 (d, *J* = 7.9 Hz, 1H), 11.66 (s, 1H). ^13^C NMR (150 MHz, DMSO-d_6_): 105.3 (CH), 111.8 (CH), 113.7 (C_quat_), 120.2 (CH), 121.9 (CH), 122.4 (CH), 125.3 (C_quat_), 128.2 (CH), 137.0 (C_quat_), 157.0 (CH), 162.7 (C_quat_), 163.5 (C_quat_). HR-MS (ESI) (*m*/*z*) calcd for (C_12_H_10_N_4_ + H)^+^: 211.0978; Found: 211.0977.

### 3.4. Synthesis of Scalaridine A (***6***)

#### 3.4.1. Synthesis of 3,5-Bis(5-methoxy-1H-indol-3-yl)pyridine (**5**)

[Pd(PPh_3_)_4_] (57.0 mg, 0.0490 μmol, 5 mol %) and 3-iodo-5-methoxy-1-tosyl-1*H*-indole (**1e**) (0.427 g, 1.00 mmol) were placed in a dry screw-cap Schlenk vessel with a septum and a magnetic stir bar under nitrogen atmosphere and were suspended in dry 1,4-dioxane (5.00 mL). The solution was degassed with nitrogen for 10 min. Dry triethylamine (1.40 mL, 10.0 mmol) and 4,4,5,5-tetramethyl-1,3,2-dioxaborolane (0.23 mL, 1.60 mmol) were added before the reaction mixture was stirred at 80 °C (preheated oil bath) for 4 h. After cooling to room temp methanol (7.00 mL), cesium carbonate (0.810 g, 2.50 mmol), and 3,5-dibromopyridine (0.120 g, 0.500 mmol) were added and the suspension was stirred at 60 °C (preheated oil bath) for 18 h. Thereafter, the reaction mixture was again cooled to room temp and potassium hydroxide (0.140 mg, 2.50 mmol) was added, the suspension was stirred at 100 °C (preheated oil bath) for 3 h. The reaction mixture was cooled to room temp and the crude product was adsorbed onto Celite^®^ and purified by flash chromatography on silica gel (eluent: dichloromethane/methanol/ammonia (aqueous 25%) 100:1:1). The obtained solid was suspended in diethyl ether, filtered, and dried in vacuo at 60 °C for 1 day. Compound **5** (0.120 g, 64%) was isolated as a beige solid, Mp 218–219 °C (107–111 °C [42]). R_f_ (dichloromethane/methanol/ammonia (aqueous 25%) 100:1:1): 0.37. 

^1^H NMR (300 MHz, DMSO-d_6_): *δ* 3.82 (s, 6H), 6.86 (dd, *J* = 8.8, 2.4 Hz, 2H), 7.38 (d, *J* = 2.4 Hz, 2H), 7.40 (d, *J* = 8.9 Hz, 2H), 7.85 (d, *J* = 2.7 Hz, 2H), 8.24 (t, *J* = 2.1 Hz, 1H), 8.77 (d, *J* = 2.7 Hz, 2H), 11.39 (s, 2H). ^13^C NMR (75 MHz, DMSO-d_6_): *δ* 55.4 (CH_3_), 100.7 (CH), 111.6 (CH), 112.1 (C_quat_), 112.8 (CH), 125.0 (CH), 125.2 (C_quat_), 130.2 (CH), 131.8 (C_quat_), 132.0 (C_quat_), 143.9 (CH), 154.2 (C_quat_). MS (EI) (*m*/*z*): 370 (26), 369 ([M]^+^, 100), 326 (13), 184 ([C_12_H_10_NO]^+^, 15), 177 (12), 163 (13), 155 ([C_11_H_8_O]^+^, 14), 141 (26). HR-MS (ESI) (*m*/*z*) calcd. for (C_23_H_19_N_3_O_2_ + H)^+^: 370.1550; Found: 370.1551.

#### 3.4.2. Synthesis of 3,3′-(Pyridine-3,5-diyl)bis(1H-indol-5-ol), Scalaridine A (**6**) by Demethylation of Compound **5**

3,5-Bis(5-methoxy-1*H*-indol-3-yl)pyridine (**5**) (85.0 mg, 0.230 mmol) was placed in a dry screw-cap Schlenk vessel with a septum and a magnetic stir bar under nitrogen atmosphere and was dissolved in acetic acid (2.00 mL). After hydrobromic acid (2.00 mL, aq, 48%) were added the suspension was stirred at 120 °C (preheated oil bath) for 16 h. After cooling to room temp, the solvent was evaporated under reduced pressure. The residue was diluted in ammonia (10.0 mL, aqueous 25%) and stirred at room temp for 1 h. The crude product was extracted with ethyl acetate (3 × 100 mL), adsorbed onto Celite^®^ and purified by flash chromatography on silica gel (eluent: ethyl acetate). Compound **6** (53 mg, 68%) was obtained as a yellow solid, Mp 319–320 °C (319–320 °C [42]). R_f_ (ethyl acetate): 0.34.

^1^H NMR (300 MHz, DMSO-d_6_): *δ* 6.71 (dd, *J* = 8.6, 2.2 Hz, 2H), 7.23 (d, *J* = 2.2 Hz, 2H), 7.30 (d, *J* = 8.7 Hz, 2H), 7.78 (d, *J* = 2.7 Hz, 2H), 8.18 (t, *J* = 2.2 Hz, 1H), 8.69 (d, *J* = 2.1 Hz, 2H), 8.81 (s, 2H), 11.26 (d, *J* = 2.7 Hz, 2H). ^13^C NMR (75 MHz, DMSO-d_6_): *δ* 102.6 (CH), 111.1 (CH), 112.0 (CH), 112.6 (C_quat_), 124.5 (CH), 125.7 (C_quat_), 130.0 (CH), 130.6 (C_quat_), 133.0 (C_quat_), 144.5 (CH), 151.1 (C_quat_). MS (ESI) (*m*/*z*): 342 (22), 341 ([M]^+^, 100), 312 (14). HR-MS (ESI) (*m*/*z*) calcd. for (C_21_H_15_N_3_O_2_ + H)^+^: 342.1237; Found: 342.1240. 

## 4. Conclusions

The Masuda borylation–Suzuki coupling sequence is a practical and catalyst-efficient tool for the synthesis of bi(hetero)aryls and (hetero)aryl-bridged bisindoles. Here, we showcased the concise two-step syntheses of the naturally occurring brominated marine alkaloids meridianins C, D, and F, as well as meridianin G and the marine bisindole scalaridine A. The operational simplicity and concision of the MBSC sequence is well suited for the rapid synthesis of libraries of related bi(hetero)aryls and corresponding bridged systems with biological activity, which are currently underway.

## Data Availability

All data of this work are included in the manuscript and the Supplementary Materials.

## Supplementary Materials

The following are available online at https://www.mdpi.com/article/10.3390/molecules27072233/s1, Supporting Information: Synthetic and analytic details, and ^1^H and ^13^C NMR spectra of compounds **1–6**, comparison of experimental NMR data with NMR data of the isolated natural products.

## Sample Availability

The data presented in this study are available on request from the corresponding author.

## References

[1] Proksch P., Putz A., Ortlepp S., Kjer J., Bayer M. (2010). Bioactive natural products from marine sponges and fungal endophytes. Phytochem. Rev..

[2] Carroll A.R., Copp B.R., Davis R.A., Keyzers R.A., Prinsep M.R. (2021). Marine natural products. Nat. Prod. Rep..

[3] Debbab A., Aly A.H., Lin W.H., Proksch P. (2010). Bioactive Compounds from Marine Bacteria and Fungi. Microb. Biotechnol..

[4] Kobayashi J.I., Murayama T., Ishibashi M., Kosuge S., Takamatsu M., Ohizumi Y., Kobayashi H., Ohta T., Nozoe S., Takuma S. (1990). Hyrtiosins A and B, new indole alkaloids from the Okinawan marine sponge Hyrtios erecta. Tetrahedron.

[5] Tsujii S., Rinehart K.L., Gunasekera S.P., Kashman Y., Cross S.S., Lui M.S., Pomponi S.A., Diaz M.C. (1988). Topsentin, bromotopsentin, and dihydrodeoxybromotopsentin: Antiviral and antitumor bis(indolyl) imidazoles from Caribbean deep-sea sponges of the family Halichondriidae. Structural and synthetic studies. J. Org. Chem..

[6] Rinehart K.L., Kobayashi J., Harbour G.C., Hughes R.G., Mizsak S.A., Scahill T.A. (1984). Eudistomins C, E, K, and L, potent antiviral compounds containing a novel oxathiazepine ring from the Caribbean tunicate *Eudistoma olivaceum*. J. Am. Chem. Soc..

[7] Bobzin S.C., Faulkner D.J. (1991). Aromatic alkaloids from the marine sponge *Chelonaplysilla* sp.. J. Org. Chem..

[8] Sakemi S., Sun H.H. (1991). Nortopsentins A, B, and C. Cytotoxic and antifungal imidazolediylbis[indoles] from the sponge Spongosorites ruetzleri. J. Org. Chem..

[9] Jiang B., Smallheer J.M., Amaral-Ly C., Wuonola M.A. (1994). Total Synthesis of (±)-Dragmacidin: A Cytotoxic Bis(indole)alkaloid of Marine Origin. J. Org. Chem..

[10] Bifulco G., Bruno I., Minale L., Riccio R., Calignano A., Debitus C. (1994). (±)-Gelliusines A and B, Two Diastereomeric Brominated Tris-indole Alkaloids from a Deep Water New Caledonian Marine Sponge (*Gellius* or *Orina* sp.). J. Nat. Prod..

[11] Franco L.H., de Kier Joffé E.B., Puricelli L., Tatian M., Seldes A.M., Palermo J.A. (1998). Indole alkaloids from the tunicate *Aplidiummeridianum*. J. Nat. Prod..

[12] Seldes A., Rodriguez Brasco M., Hernández Franco L., Palermo J. (2007). Identification of two meridianins from the crude extract of the tunicate *Aplidium meridianum* by tandem mass spectrometry. Nat. Prod. Res..

[13] Gompel M., Leost M., de Kier Joffé E.B., Puricelli L., Franco L.H., Palermo J., Meijer L. (2004). Meridianins, a new family of protein kinase inhibitors isolated from the ascidian *Aplidium meridianum*. Bioorg. Med. Chem. Lett..

[14] Lee Y.J., Lee D.G., Rho H.S., Krasokhin V.B., Shin H.J., Lee J.S., Lee H.S. (2013). Cytotoxic 5-Hydroxyindole Alkaloids from the Marine Sponge *Scalarispongia* sp.. J. Heterocycl. Chem..

[15] Murata M., Watanabe S., Masuda Y. (1997). Novel Palladium(0)-Catalyzed Coupling Reaction of Dialkoxyborane with Aryl Halides: Convenient Synthetic Route to Arylboronates. J. Org. Chem..

[16] Murata M., Oyama T., Watanabe S., Masuda Y. (2000). Palladium-Catalyzed Borylation of Aryl Halides or Triflates with Dialkoxyborane: A Novel and Facile Synthetic Route to Arylboronates. J. Org. Chem..

[17] Baudoin O., Guénard D., Guéritte F. (2000). Palladium-Catalyzed Borylation of Ortho-Substituted Phenyl Halides and Application to the One-Pot Synthesis of 2,2′-Disubstituted Biphenyls. J. Org. Chem..

[18] Baudoin O., Cesario M., Guénard D., Guéritte F. (2002). Application of the Palladium-Catalyzed Borylation/Suzuki Coupling (BSC) Reaction to the Synthesis of Biologically Active Biaryl Lactams. J. Org. Chem..

[19] Penhoat M., Levacher V., Dupas G. (2003). Novel Extension of Meyers’ Methodology: Stereoselective Construction of Axially Chiral 7,5-Fused Bicyclic Lactams. J. Org. Chem..

[20] Broutin P.-E., Čerňa I., Campaniello M., Leroux F., Colobert F. (2004). Palladium-Catalyzed Borylation of Phenyl Bromides and Application in One-Pot Suzuki−Miyaura Biphenyl Synthesis. Org. Lett..

[21] Abreu A.S., Ferreira P.M.T., Queiroz M.-J.R.P., Ferreira I.C.F.R., Calhelha R.C., Estevinho L.M. (2005). Synthesis of β-Benzo[b]thienyldehydrophenylalanine Derivatives by One-Pot Palladium-Catalyzed Borylation and Suzuki Coupling (BSC) and Metal-Assisted Intramolecular Cyclization–Studies of Fluorescence and Antimicrobial Activity. Eur. J. Org. Chem..

[22] Merkul E., Schäfer E., Müller T.J.J. (2011). Rapid synthesis of bis(hetero)aryls by one-pot Masuda borylation-Suzuki coupling sequence and its application to concise total syntheses of meridianins A and G. Org. Biomol. Chem..

[23] Tasch B.O.A., Merkul E., Müller T.J.J. (2011). One-Pot Synthesis of Diazine-Bridged Bisindoles and Concise Synthesis of the Marine Alkaloid Hyrtinadine A. Eur. J. Org. Chem..

[24] Tasch B.O.A., Antovic D., Merkul E., Müller T.J.J. (2013). One-Pot Synthesis of Camalexins and 3,3′-Biindoles by the Masuda Borylation–Suzuki Arylation (MBSA) Sequence. Eur. J. Org. Chem..

[25] Fresneda P.M., Molina P., Delgado S., Bleda J.A. (2000). Synthetic studies towards the 2-aminopyrimidine alkaloids variolins and meridianins from marine origin. Tetrahedron Lett..

[26] Fresneda P.M., Molina P., Bleda J.A. (2001). Synthesis of the indole alkaloids meridianins from the tunicate *Aplidium meridianum*. Tetrahedron.

[27] Echalier A., Bettayeb K., Ferandin Y., Lozach O., Clément M., Valette A., Liger F., Marquet B., Morris J.C., Endicott J.A. (2008). Meriolins (3-(Pyrimidin-4-yl)-7-azaindoles): Synthesis, Kinase Inhibitory Activity, Cellular Effects, and Structure of a CDK2/Cyclin A/Meriolin Complex. J. Med. Chem..

[28] Sperry J. (2011). A concise synthesis of meridianin F. Tetrahedron Lett..

[29] Tibiletti F., Simonetti M., Nicholas K.M., Palmisano G., Parravicini M., Imbesi F., Tollari S., Penoni A. (2010). One-pot synthesis of meridianins and meridianin analogues via indolization of nitrosoarenes. Tetrahedron.

[30] Parsons T.B., Ghellamallah C., Male L., Spencer N., Grainger R.S. (2011). Regioselective dibromination of methyl indole-3-carboxylate and application in the synthesis of 5,6-dibromoindoles. Org. Biomol. Chem..

[31] Walker S.R., Czyz M.L., Morris J.C. (2014). Concise Syntheses of Meridianins and Meriolins Using a Catalytic Domino Amino-Palladation Reaction. Org. Lett..

[32] Jiang B., Yang C.-G. (2000). Synthesis of Indolylpyrimidines via Cross-Coupling of Indolylboronic Acid with Choropyrimidines: Facile Synthesis of Meridianin D. Heterocycles.

[33] Dorsch D., Sirrenberg C., Müller T.J.J. (2007). Preparation of 4-(Pyrrolopyridinyl)pyrimidinyl-2-amines as Antitumor Agents. Patent.

[34] Dorsch D., Wuchrer M., Burgdorf L.T., Sirrenberg C., Esdar C., Müller T.J.J., Merkul E. (2008). Preparation of Pyrrolopyridinylpyrimidinylamines as Inhibitors of Cell Proliferation. Patent.

[35] Dorsch D., Sirrenberg C., Müller T.J.J., Merkul E. (2009). Preparation of 3-(4-pyridinyl)-1H-pyrrolo[2,3-b]pyridines as Anti-Tumor Agents. Patent.

[36] Dorsch D., Sirrenberg C., Müller T.J.J., Merkul E., Karapetyan G. (2012). 7-Azaindole Derivatives as Kinase Inhibitors and Their Preparation and Use in the Treatment of Tumors. Patent.

[37] Drießen D., Stuhldreier F., Frank A., Stark H., Wesselborg S., Stork B., Müller T.J.J. (2019). Novel meriolin derivatives as rapid apoptosis inducers. Bioorg. Med. Chem..

[38] Müller T.J.J. (2006). Sequentially Palladium-Catalyzed Processes. Top. Organomet. Chem..

[39] Lessing T., Müller T.J.J. (2015). Sequentially Palladium-Catalyzed Processes in One-Pot Syntheses of Heterocycles. Appl. Sci..

[40] Rehberg N., Sommer G.A., Drießen D., Kruppa M., Adeniyi E.T., Chen S., Wang L., Wolf K., Tasch B.O.A., Ioerger T.R. (2020). Nature-Inspired (di)Azine-Bridged Bisindole Alkaloids with Potent Antibacterial In Vitro and In Vivo Efficacy against Methicillin-Resistant Staphylococcus aureus. J. Med. Chem..

[41] Karpov A.S., Merkul E., Rominger F., Müller T.J.J. (2005). Concise Syntheses of Meridianins by Carbonylative Alkynylation and a Four-Component Pyrimidine Synthesis. Angew. Chem. Int. Ed..

[42] Kim S.H., Sperry J. (2015). Synthesis of scalaridine A. Tetrahedron Lett..

[43] Ansari N.H., Söderberg B.C.G. (2016). Short syntheses of the indole alkaloids alocasin A, scalaridine A, and hyrtinadine A-B. Tetrahedron.

[44] Ranjani G., Nagarajan R. (2017). Insight into Copper Catalysis: In Situ Formed Nano Cu_2_O in Suzuki–Miyaura Cross-Coupling of Aryl/Indolyl Boronates. Org. Lett..

[45] Witulski B., Buschmann N., Bergsträßer U. (2000). Hydroboration and Suzuki–Miyaura Coupling Reactions with the Electronically Modulated Variant of an Ynamine: The Synthesis of (E)-β-Arylenamides. Tetrahedron.

[46] Kim S.H., Sperry J. (2015). Synthesis of Alocasin A. J. Nat. Prod..

[47] Hilbert G.E., Johnson T.B. (1930). Researches on pyrimidines. CXIII. An improved method for the synthesis of cytosine^1^. J. Am. Chem. Soc..

[48] Kofler A., Kolšek J. (1970). Beitrag zur mikroskopischen Identifizierung organischer Stoffe nach L. Kofler. IV. Microchim. Acta.

