# Supplementary material for: Concise Syntheses of Marine (Bis)indole Alkaloids Meridianin C, D, F, and G and Scalaridine A via One-Pot Masuda Borylation-Suzuki Coupling Sequence
Source: Molecules. 2022 Mar 30;27(7):2233. doi: 10.3390/molecules27072233 (PMC9000334; doi:10.3390/molecules27072233)
Supplement: Supplementary file 1 [file molecules-27-02233-s001.zip › molecules-1641406-supplementary.pdf]

Supporting Information

Concise syntheses of marine (bis)indole alkaloids meridianin C, D, F, and G and scalaridine A via one-pot Masuda borylation-Suzuki coupling sequence

Marco Kruppa,[1] Gereon A. Sommer,[1] and Thomas J. J. Müller [1,*]

[1] Institut für Organische Chemie und Makromolekulare Chemie, Heinrich-Heine-Universität Düsseldorf, Universitätsstrasse 1, D-40225 Düsseldorf, Germany; ThomasJJ.Mueller@uni-duesseldorf.de

* Correspondence: ThomasJJ.Mueller@uni-duesseldorf.de; Tel.: +49 (0)211 81 12298 (TJJM)

Table of Contents

S1 General considerations 2

S2 Syntheses 3

S2.1 Synthesis of Meridianins **3** 3

S2.2 Synthesis of 3,5-bis(5-methoxy-1*H*-indol-3-yl)pyridine (**5**) 7

S2.3 Synthesis of 3,3'-(pyridine-3,5-diyl)bis(1*H*-indol-5-ol) (scalaridine A) (**6**) 8

S3 Comparison of experimental NMR data with NMR data of the isolated natural products..... 9

S4 NMR spectra 12

S5 References 18

S1 General considerations

All cross-coupling reactions were carried out in oven-dried Schlenk tubes under nitrogen atmosphere. By using *MBraun system MB-SPS-800* dry 1,4-dioxane was obtained. Dry triethylamine was stored in a Schlenk flask with potassium hydroxide pallets under nitrogen atmosphere.

The used *N*-protected 3-iodo-1*H*-indoles **1** have been prepared using a literature known one-pot process [1-3]. 4-chloropyrimidine-2-amine (**2**) has been synthesized by suspending 2,4-dichloroprimidine in ammonia (aq, 5%) for 2 d [4]. All other used chemicals were purchased at *Sigma-Aldrich Chemie GmbH, Acros Organics, ABCR GmbH & CO. KG, Alfa Aesar GmbH* and *Merck Serono KGaA* and used as supplied.

For purification of the reaction mixtures a flash chromatography was performed on silica gel 60 (0.015-0.040 mm) from *Macherey-Nagel GmbH & Co. KG* under a pressure of 2 bar. Therefore, the crude reaction mixtures were adsorbed on Celite® 545 (0.02-0.10 mm) from *Macherey-Nagel GmbH & Co. KG*. For TLC Silica gel 60 F_{254} 6 x 6 cm aluminum sheets by *Macherey-Nagel GmbH & Co*. KG were used. The spots were detected with UV light at 254 and 365 nm. ^{1}H, ^{13}C and 135-DEPT NMR spectra were recorded on *Bruker Avance III 300* and *Bruker Acance III 600* spectrometer. DMSO-d_6 was used as deuterated solvents. For ^{1}H spectra the residual proton signal of the deuterated solvent was locked as internal standard

(DMSO-d_6, δ_H 2.50, δ_C 39.5). The multiplicities of signals were abbreviated as follows: s: singulet, d: doublet, t: triplet, dd: doublet of doublets. The types of carbon atoms were abbreviated as follows: CH_3: primary carbon atom, CH_2: secondary carbon atom, CH: tertiary carbon atom and C_{quat}: quartary carbon atom. For determination 135-DEPT NMR spectra was used. Mass spectra were measured on *Varian MAT 311 A*. All Peaks with an intensity of >10 % corresponding to the base peak were stated. The melting points (uncorrected) were measured on *Rechert-Jung S3 Thermovar* [5]. Elementary analysis was carried out in the micro analytical laboratory of Institut für Pharmazeutische und Medizinische Chemie der Heinrich-Heine-Universität, Düsseldorf.

S2 Syntheses

S2.1 Synthesis of Meridianins 3

S2.1.1 Synthesis of 4-(6-bromo-1*H*-indol-3-yl)pyrimidin-2-amine, meridianin D (3a)

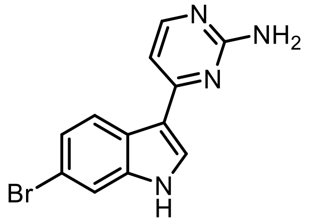

[289.14]
$C_{12}H_9BrN_4$

[Pd(PPh_3)$_4$] (19.0 mg, 0.0190 mmol, 3 mol %) and *tert*-butyl 6-bromo-3-iodo-1*H*-indole-1-carboxylate (**1a**) (278 mg, 0.660 mmol) were placed in a dry screw-cap Schlenk vessel with a septum and a magnetic stir bar under nitrogen atmosphere and suspended in dry 1,4-dioxane (5.00 mL). After the addition of dry triethylamine (0.920 mL, 6.59 mmol) and 4,4,5,5-tetramethyl-1,3,2-dioxaborolane (0.140 mL, 0.990 mmol) the mixture was stirred at 80 °C (preheated oil bath) for 4 h (monitored by TLC). After cooling to room temperature, methanol (5.00 mL), cesium carbonate (0.540 g, 1.65 mmol) and 4-chloropyrimidine-2-amin (**2**) (0.0850 g, 0.660 mmol) were added. Then, the mixture was stirred at 100 °C (preheated oil bath) for 18 h. After cooling to room temperature, the solution was diluted with dichloromethane and adsorbed onto Celite®. The solvent was removed under reduced pressure and the product was purified by flash chromatography on silica gel (eluent: dichloromethane/methanol/ammonia (aq, 25%) 100:5:1). The obtained solid was dried under vacuo at 60 °C for 3 to 4 days. Compound **3a** (43 mg, 25%) was obtained as a yellow solid.

R_f (dichloromethane/methanol/ammonia (aq, 25%) 100:5:1): 0.32.

Mp: 211-213 °C (217-221 °C [6]).

^{1}H NMR (300 MHz, DMSO-d$_6$): δ 6.46 (s, 2H), 7.00 (d, *J* = 5.3 Hz, 1H), 7.23 (dd, *J* =8.5, 1.9 Hz, 1H), 7.62 (d, *J* = 1.8 Hz, 1H), 8.11 (d, *J* = 5.3 Hz, 1H), 8.23 (d, *J* = 2.9 Hz, 1H), 8.56 (d, *J* = 8.5 Hz, 1H), 11.78 (s, 1H).

^{13}C NMR (75 MHz, DMSO-d$_6$): δ 105.2 (CH), 113.2 (C_{quat}), 113.3 (C_{quat}), 113.7 (CH), 124.5 (CH), 124.6 (CH), 127.0 (C_{quat}), 129.6 (CH), 135.7 (C_{quat}), 157.1 (CH), 162.2 (C_{quat}), 163.5 (C_{quat}).

HR-MS (ESI) (*m*/*z*) calcd for ($C_{12}H_9{}^{79}BrN_4$+H)$^+$: 289.0088; Found: 289.0081.

S2.1.2 General procedure I (GPI) Synthesis of meridianin C (3e), meridianin F (3f) and meridianin G (3g) via MBSC sequence

[$Pd(PPh_3)_4$] (19.0 mg, 0.0190 mmol, 3 mol %) and the 3-iodo-1-tosyl-1*H*-indole **1** (0.660 mmol) were placed under nitrogen atmosphere in a dry screw-cap Schlenk vessel with a septum and a magnetic stir bar and were suspended in dry 1,4-dioxane (5.00 mL). After the addition of dry triethylamine (0.920 mL, 6.59 mmol) and 4,4,5,5-tetramethyl-1,3,2-dioxaborolane (0.140 mL, 0.990 mmol) the mixture was stirred at 80 °C (preheated oil bath) for 4 h (monitored by TLC). After cooling to room temperature, methanol (5.00 mL), cesium carbonate (0.540 g, 1.65 mmol) and 4-chloropyrimidine-2-amin (**2**) (0.0850 g, 0.660 mmol) were added. Then, the mixture was stirred at 100 °C (preheated oil bath) for 15 h. The solution was again cooled to room temperature before potassium hydroxide (0.110 g, 1.98 mmol) was added. After the mixture was allowed to cool down to room temperature it was diluted with dichloromethane and adsorbed onto Celite®. The solvent was removed under reduced pressure and the product was purified by flash chromatography on silica gel (eluent: dichloromethane/methanol/ammonia (aq, 25%) (100:5:1)). The obtained solid was dried in vacuo at 60 °C for 4 days (Table S1).

Table S1: Experimental details for the synthesis of meridianins **3**.

**Entry**	**3-Iodo-1-tosyl-1*H*-indole 1 [g] ([mmol])**	**Meridianin 3**	**Yield [%] [g] ([mmol])**
**1**	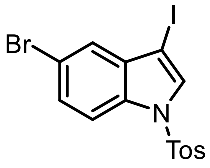 [0.314 mg, 0.660 mmol]	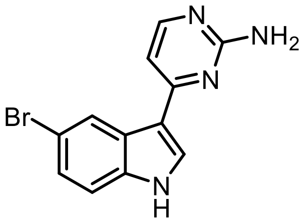 meridianin C, **3e**	48% [91 mg, 0.318 mmol]
**2**	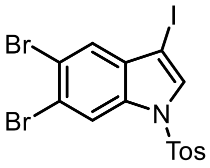 [0.330 g, 0.660 mmol]	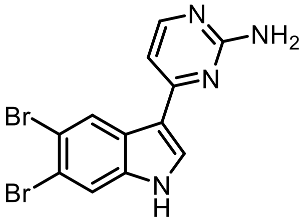 meridianin F, **3f**	66% [160 mg, 0.435 mmol]
**3**	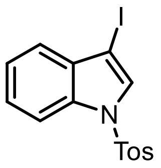 [0.263 g, 0.660 mmol]	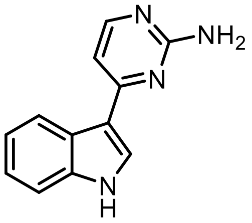 meridianin G, **3g**	80% [111 mg, 0.528 mmol]

S2.1.2.1 Analytic data

4-(5-bromo-1*H*-indol-3-yl)pyrimidin-2-amine, meridianin C (3e)

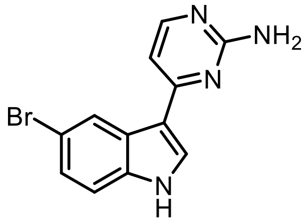

[289.14]
$C_{12}H_9BrN_4$

According to the general procedure starting from 5-bromo-3-iodo-1-tosyl-1*H*-indole (**1c**) compound **3e** (91 mg, 48%) was isolated as a yellow solid.

R_f (dichloromethane/methanol/ammonia (aq, 25%) 100:5:1): 0.25.

Mp: 208-210 °C (238-240 °C [7]).

^{1}H NMR (300 MHz, DMSO-d$_6$): δ 6.50 (s, 2H), 7.01 (d, *J* = 5.4 Hz, 1H), 7.29 (dd, *J* = 8.6, 2.0 Hz, 1H), 7.41 (d, *J* = 8.6 Hz, 1H), 8.11 (d, *J* = 5.3 Hz, 1H), 8.26 (d, *J*= 2.9 Hz, 1H), 8.76 (d, *J* = 2.0 Hz, 1H), 11.86 (s, 1H).

^{13}C NMR (75 MHz, DMSO-d$_6$): δ 105.2 (CH), 113.2 (C_{quat}), 113.3 (C_{quat}), 113.7 (CH), 124.5 (CH), 124.6 (CH), 127.0 (C_{quat}), 129.6 (CH), 135.7 (C_{quat}), 157.0 (CH), 162.2 (C_{quat}), 163.5 (C_{quat}).

HR-MS (ESI) (*m*/*z*) calcd for $(C_{12}H_9{}^{79}BrN_4+H)^+$: 289.0088; Found: 289.0083.

Anal calcd for $C_{12}H_9BrN_4$ (289.1): C 49.85, H 3.14, N 19.38; Found: C 50.04, H 3.14, N 19.09.

4-(5,6-dibromo-1*H*-indol-3-yl)pyrimidin-2-amine, meridianin F (3f)

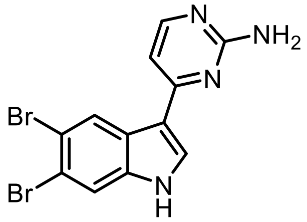

[368.03]
$C_{12}H_8BrN_4$

According to the general procedure starting from 5,6-dibromo-3-iodo-1-tosyl-1*H*-indole (**1b**) compound **3f** (0.160 g, 66%) was obtained as a colorless solid.

R_f (dichloromethane/methanol/ammonia (aq, 25%) 100:5:1): 0.22.

Mp: 290-292 °C (167-169 °C [8]).

^{1}H NMR (300 MHz, DMSO-d$_6$): δ 6.56 (s, 2H), 7.02 (d, *J* = 5.3 Hz, 1H), 7.85 (s, 1H), 8.14 (d, *J* = 5.3 Hz, 1H), 8.31 (d, *J* = 1.7 Hz, 1H), 8.97 (s, 1H), 11.94 (s, 1H).

^{13}C NMR (75 MHz, DMSO-d$_6$): δ 105.1 (CH), 113.2 (C_{quat}), 114.9 (C_{quat}), 116.1 (C_{quat}), 116.5 (CH), 126.1 (C_{quat}), 126.5 (CH), 130.4 (CH), 136.7 (C_{quat}), 157.3 (CH), 161.8 (C_{quat}), 163.5 (C_{quat}).

HR-MS (ESI) (*m*/*z*) calcd for $(C_{12}H_8{}^{79}Br_2N_4+H)^+$: 366.9188; Found: 366.9171.

Anal calcd for $C_{12}H_8Br_2N_4$ (368.03): C 39.16, H 2.19, N 15.22; Found: C 39.36, H 2.36, N 14.93.

4-(1*H*-indol-3-yl)pyrimidin-2-amine, meridianin G (3g)

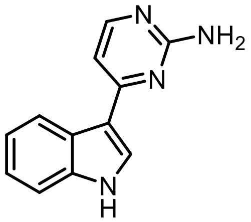

[210.24]
$C_{12}H_{10}N_4$

According to the general procedure starting from 3-iodo-1-tosyl-1*H*-indole (**1d**) compound **3g** (111 mg, 80%) was isolated as a colorless solid.

R$_f$ (dichloromethane/methanol/ammonia (aq, 25%) 100:5:1): 0.26.

Mp: 190-191 °C (195-197 °C [9]).

^{1}H NMR (600 MHz, DMSO-d$_6$): δ 6.41 (s, 2H), 7.01 (d, *J* = 5.3 Hz, 1H), 7.12 (td, *J* = 7.4, 6.9, 1.1 Hz, 1H), 7.17 (ddd, *J* = 8.1, 6.9, 1.3 Hz, 1H), 7.44 (d, *J* = 8.0 Hz, 1H), 8.10 (d, *J* = 5.3 Hz, 1H), 8.19 (d, *J* = 2.9 Hz, 1H), 8.58 (d, *J* = 7.9 Hz, 1H), 11.66 (s, 1H).

^{13}C NMR (150 MHz, DMSO-d$_6$): 105.3 (CH), 111.8 (CH), 113.7 (C_{quat}), 120.2 (CH), 121.9 (CH), 122.4 (CH), 125.3 (C_{quat}), 128.2 (CH), 137.0 (C_{quat}), 157.0 (CH), 162.7 (C_{quat}), 163.5 (C_{quat}).

HR-MS (ESI) (*m*/*z*) calcd for $(C_{12}H_{10}N_4+H)^+$: 211.0978; Found: 211.0977.

S2.2 Synthesis of 3,5-bis(5-methoxy-1*H*-indol-3-yl)pyridine (5)

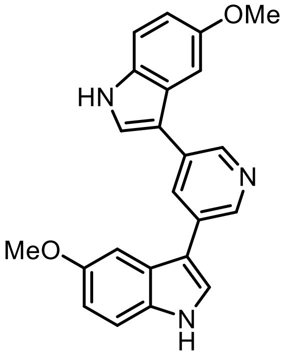

[369.42]
$C_{23}H_{19}N_3O_2$

$[Pd(PPh_3)_4]$ (57.0 mg, 0.0490 mmol, 5 mol %) and 3-iodo-5-methoxy-1-tosyl-1*H*-indole (**1e**) (0.427 g, 1.00 mmol) were placed in a dry screw-cap Schlenk vessel with a septum and a magnetic stir bar under nitrogen atmosphere and were suspended in dry 1,4-dioxane (5.00 mL). The solution was degassed with nitrogen for 10 min. Dry triethylamine (1.40 mL, 10.0 mmol) and 4,4,5,5-tetramethyl-1,3,2-dioxaborolane (0.230 mL, 1.60 mmol) were added before the reaction mixture was stirred at 80 °C (preheated oil bath) for 4 h. After cooling to room temp methanol (7.00 mL), cesium carbonate (0.810 g, 2.50 mmol) and 3,5-dibromopyridine (0.120 g, 0.500 mmol) were added and the suspension was stirred at 60 °C (preheated oil bath) for 18 h. Thereafter, the reaction mixture was again cooled to room temp and potassium hydroxide (0.140 mg, 2.50 mmol) was added and the suspension was stirred at 100 °C (preheated oil bath) for 3 h. The reaction mixture was cooled to room temp and the crude product was adsorbed onto Celite® and purified by flash chromatography on silica gel (eluent: dichloromethane/methanol/ammonia (aq, 25%) 100:1:1). The obtained solid was suspended in diethyl ether, filtered and dried in vacuo at 60 °C for 1 day. Compound **5** (0.120 g, 64%) was isolated as a beige solid.

$\mathbf{R_f}$ (dichloromethane/methanol/ammonia (aq, 25%) 100:1:1): 0.37.

Mp: 218-219 °C (107-111 °C [10]).

^{1}H NMR (300 MHz, DMSO-d$_6$): δ 3.82 (s, 6H), 6.86 (dd, J = 8.8, 2.4 Hz, 2H), 7.38 (d, J = 2.4 Hz, 2H), 7.40 (d, J = 8.9 Hz, 2H), 7.85 (d, J = 2.7 Hz, 2H), 8.24 (t, J = 2.1 Hz, 1H), 8.77 (d, J = 2.7 Hz, 2H), 11.39 (s, 2H).

^{13}C NMR (75 MHz, DMSO-d$_6$): δ 55.4 (CH_3), 100.7 (CH), 111.6 (CH), 112.1 (C_{quat}), 112.8 (CH), 125.0 (CH), 125.2 (C_{quat}), 130.2 (CH), 131.8 (C_{quat}), 132.0 (C_{quat}), 143.9 (CH), 154.2 (C_{quat}).

MS (EI) (*m*/*z*): 370 (26), 369 ($[M]^+$, 100), 326 (13), 184 ($[C_{12}H_{10}NO]^+$, 15), 177 (12), 163 (13), 155 ($[C_{11}H_8O]^+$, 14), 141 (26).

HR-MS (ESI) (*m*/*z*) calcd. for $(C_{23}H_{19}N_3O_2+H)^+$: 370.1550; Found: 370.1551.

S2.3 Synthesis of 3,3'-(pyridine-3,5-diyl)bis(1*H*-indol-5-ol) (scalaridine A) (6)

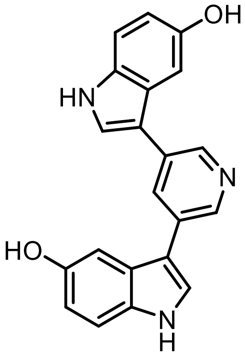

[341.37]
$C_{21}H_{15}N_3O_2$

3,5-Bis(5-methoxy-1*H*-indol-3-yl)pyridine (**5**) (85.0 mg, 0.230 mmol) was placed in a dry screw-cap Schlenk vessel with a septum and a magnetic stir bar under nitrogen atmosphere and was dissolved in acetic acid (2.00 mL). After hydrobromic acid (2.00 mL, aq, 48%) were added the suspension was stirred at 120 °C (preheated oil bath) for 16 h. After cooling to room temp, the solvent was evaporated under reduced pressure. The residue was diluted in ammonia (10.0 mL, aq, 25%) and stirred at room temp for 1 h. The crude product was extracted with ethyl acetate (3 x 100 mL), adsorbed onto Celite® and purified by flash chromatography on silica gel (eluent: ethyl acetate). Compound **6** (53.0 mg, 68%) was obtained as a yellow solid.

$\mathbf{R_f}$ (ethyl acetate): 0.34.

Mp: 319-320 °C (319-320 °C [10]).

^{1}H NMR (300 MHz, DMSO-d$_6$): δ 6.71 (dd, *J* = 8.6, 2.2 Hz, 2H), 7.23 (d, *J* = 2.2 Hz, 2H), 7.30 (d, *J* = 8.7 Hz, 2H), 7.78 (d, *J* = 2.7 Hz, 2H), 8.18 (t, *J* = 2.2 Hz, 1H), 8.69 (d, *J* = 2.1 Hz, 2H), 8.81 (s, 2H), 11.26 (d, *J* = 2.7 Hz, 2H).

^{13}C NMR (75 MHz, DMSO-d$_6$): δ 102.6 (CH), 111.1 (CH), 112.0 (CH), 112.6 (C_{quat}), 124.5 (CH), 125.7 (C_{quat}), 130.0 (CH), 130.6 (C_{quat}), 133.0 (C_{quat}), 144.5 (CH), 151.1 (C_{quat}).

MS (ESI) (*m*/*z*): 342 (22), 341 ($[M]^+$, 100), 312 (14).

HR-MS (ESI) (*m*/*z*) calcd. for $(C_{21}H_{15}N_3O_2+H)^+$: 342.1237; Found: 342.1240.

S3 Comparison of experimental NMR data with NMR data of the isolated natural products

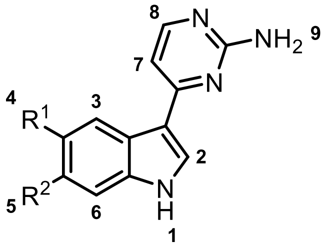

meridianin C R^1 = Br, R^2 = H (3e)		
**proton**	**experimental[a]**	**literature, isolated compound[b] [6] [4]**
**1**	s, 11.86	s, 11.85
**2**	d, 8.26	d, 8.24
**3**	d, 8.76	d, 8.75
**4**	-	-
**5**	dd, 7.29	dd, 7.28
**6**	d, 7.41	d, 7.41
**7**	d, 7.01	d, 7.00
**8**	d, 8.11	d, 8.11
**9**	s, 6.50	s, 6.48

[a] 300 MHz, DMSO-d_6, [b] 200 MHz, DMSO-d_6

meridianin D R^1 = H, R^2 = Br (3a)		
**proton**	**experimental[a]**	**literature, isolated compound[b] [6] [4]**
**1**	s, 11.78	s, 11.76
**2**	d, 8.23	d, 8.21
**3**	d, 8.56	d, 8.55
**4**	dd, 7.23	dd, 7.24
**5**	-	-
**6**	d, 7.62	d, 7.63
**7**	d, 7.00	d, 7.00
**8**	d, 8.11	d, 8.12
**9**	d, 6.46	d, 6.43

[a]300 MHz, DMSO-d_6, [b]200 MHz, DMSO-d_6

	meridianin F R^1 = Br, R^2 = Br (3f)	
**proton**	**experimental[a]**	**literature, isolated compound[b] [7] [5]**
**1**	s, 11.94	-
**2**	d, 8.31	s, 8.07
**3**	s, 8.97	s, 8.86
**4**	-	-
**5**	-	-
**6**	s, 7.85	s, 7.77
**7**	d, 7.02	d, 7.00
**8**	d, 8.14	d, 8.09
**9**	s, 6.56	s, 6.42

[a] 300 MHz, DMSO-d_6, [b] 500 MHz, CD_3OD

	meridianin G R^1 = H, R^2 = H (3g)	
**proton**	**experimental[a]**	**literature, isolated compound[b] [7] [5]**
**1**	s, 11.66	-
**2**	d, 8.19	s, 7.97
**3**	d, 8.58	dd, 8.36
**4**	td, 7.12	ddd, 7.12
**5**	ddd, 7.17	ddd, 7.15
**6**	d, 7.44	dd, 7.38
**7**	d, 7.01	d, 7.02
**8**	d, 8.10	d, 8.05
**9**	s, 6.41	-

[a] 600 MHz, DMSO-d_6, [b] 500 MHz, CD_3OD

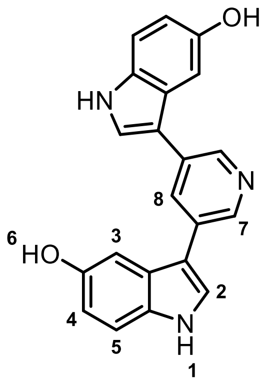

scalaridine A		
**proton**	**experimental[a]**	**literature, isolated compound[b] [8] [6]**
**1**	s, 11.26	s, 11.26
**2**	d, 7.78	s, 7.77
**3**	d, 7.23	d, 7.23
**4**	dd, 6.71	dd, 6.70
**5**	d, 7.30	d, 7.29
**6**	s, 8.81	s, 8.82
**7**	d, 8.69	s, 8.68
**8**	t, 8.18	s, 8.17

[a] 300 MHz, DMSO-d_6, [b] 500 MHz, DMSO-d_6

S4 NMR spectra

^{1}H NMR-spectrum of 4-(5-bromo-1*H*-indol-3-yl)pyrimidin-2-amine (meridianin C) (**3e**) (300 MHz, DMSO-d_6, 293 K).

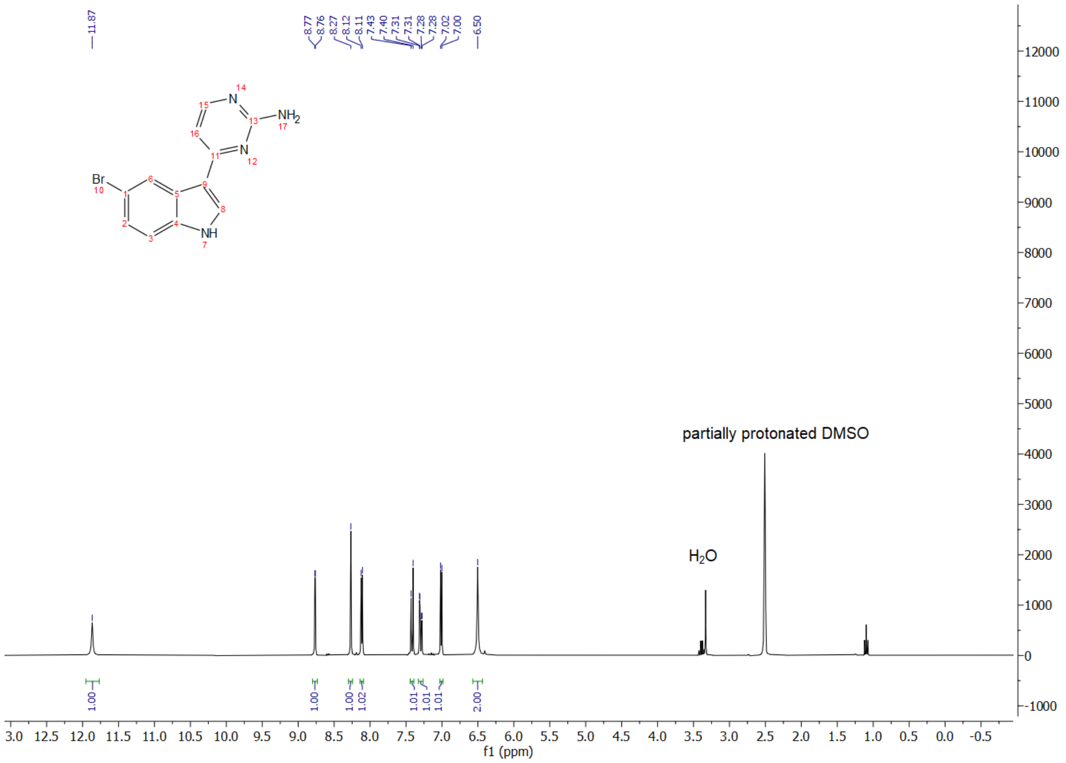

^{13}C NMR-spectrum of 4-(5-bromo-1*H*-indol-3-yl)pyrimidin-2-amine (meridianin C) (**3e**) (75 MHz, DMSO-d_6, 293 K).

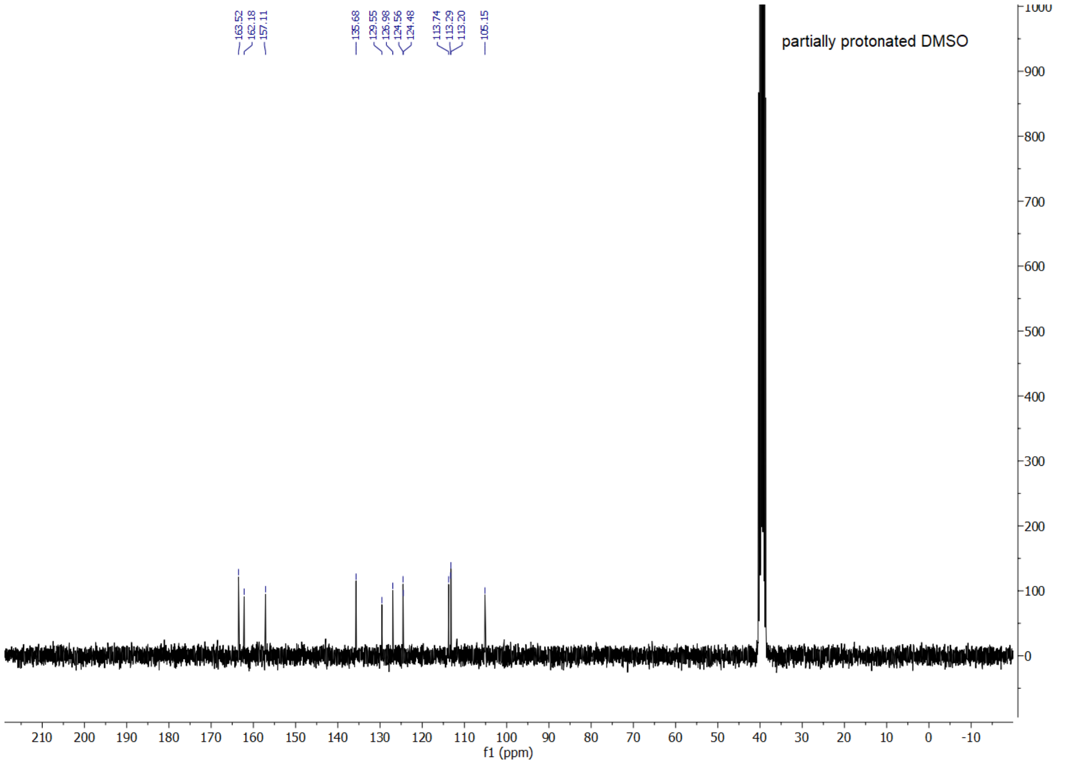

^{1}H NMR-spectrum of 4-(6-bromo-1*H*-indol-3-yl)pyrimidin-2-amine (meridianin D) (**3a**) (300 MHz, DMSO-d$_6$, 293 K).

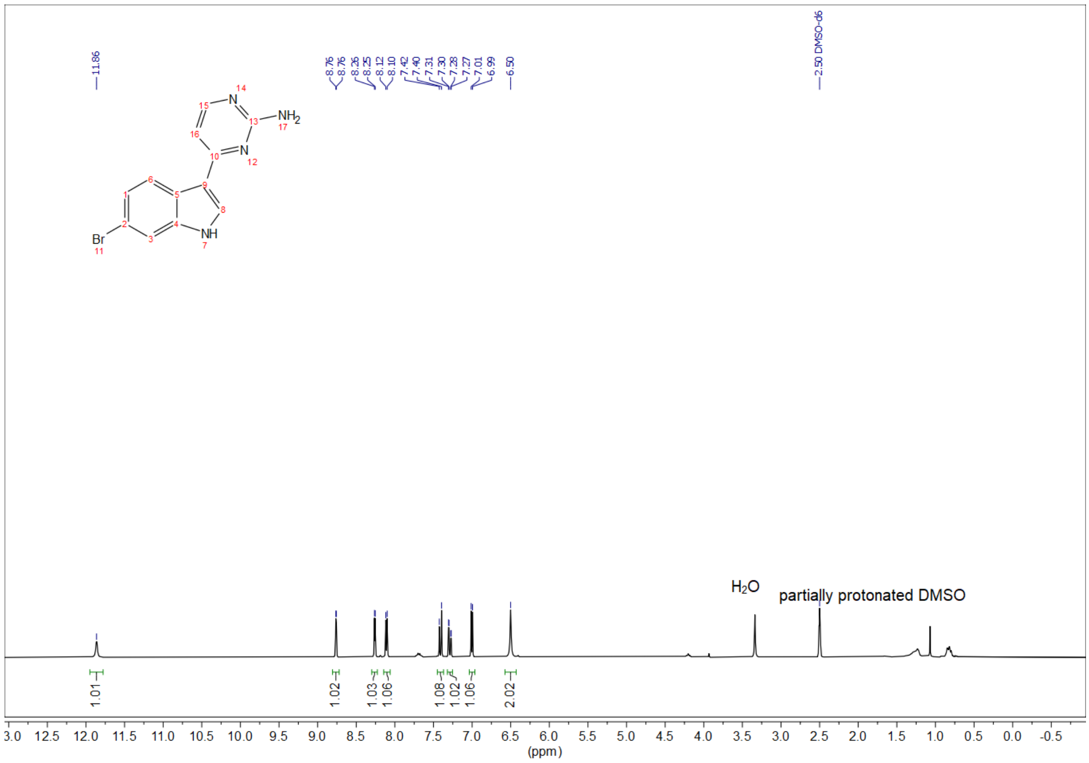

^{13}C NMR-spectrum of 4-(6-bromo-1*H*-indol-3-yl)pyrimidin-2-amine (meridianin D) (**3a**) (75 MHz, DMSO-d$_6$, 293 K).

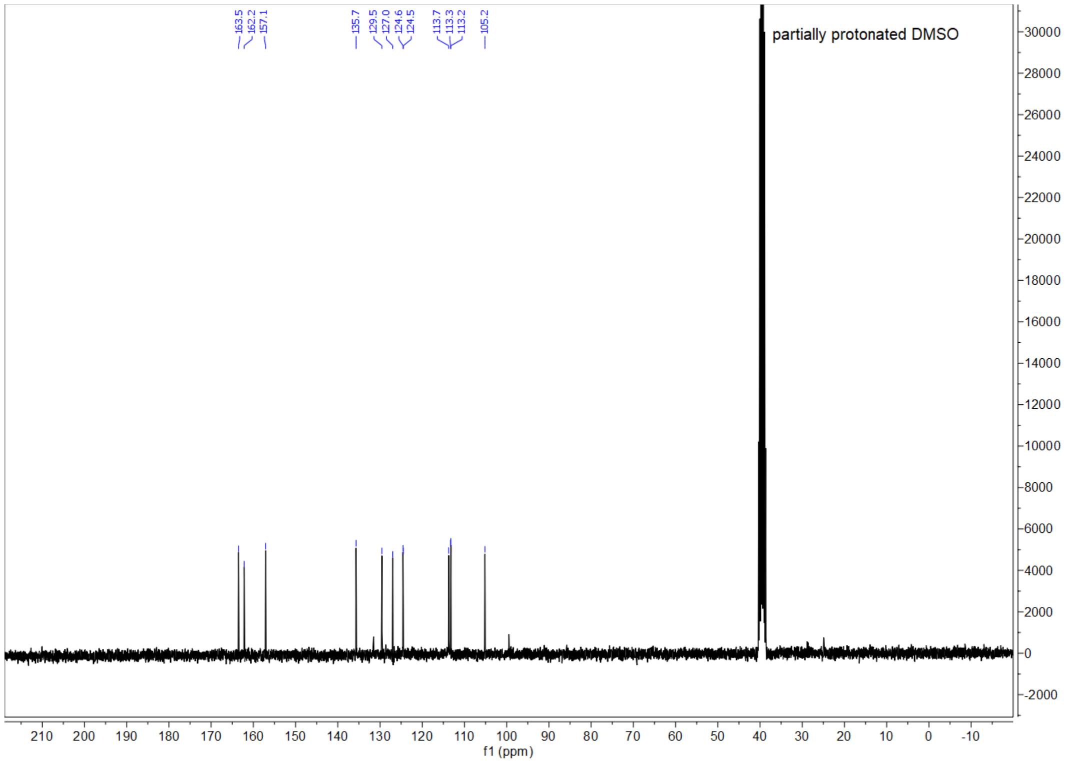

^{1}H NMR-spectrum of 4-(5,6-dibromo-1*H*-indol-3-yl)pyrimidin-2-amine (meridianin F) (**3f**) (300 MHz, DMSO-d_6, 293 K).

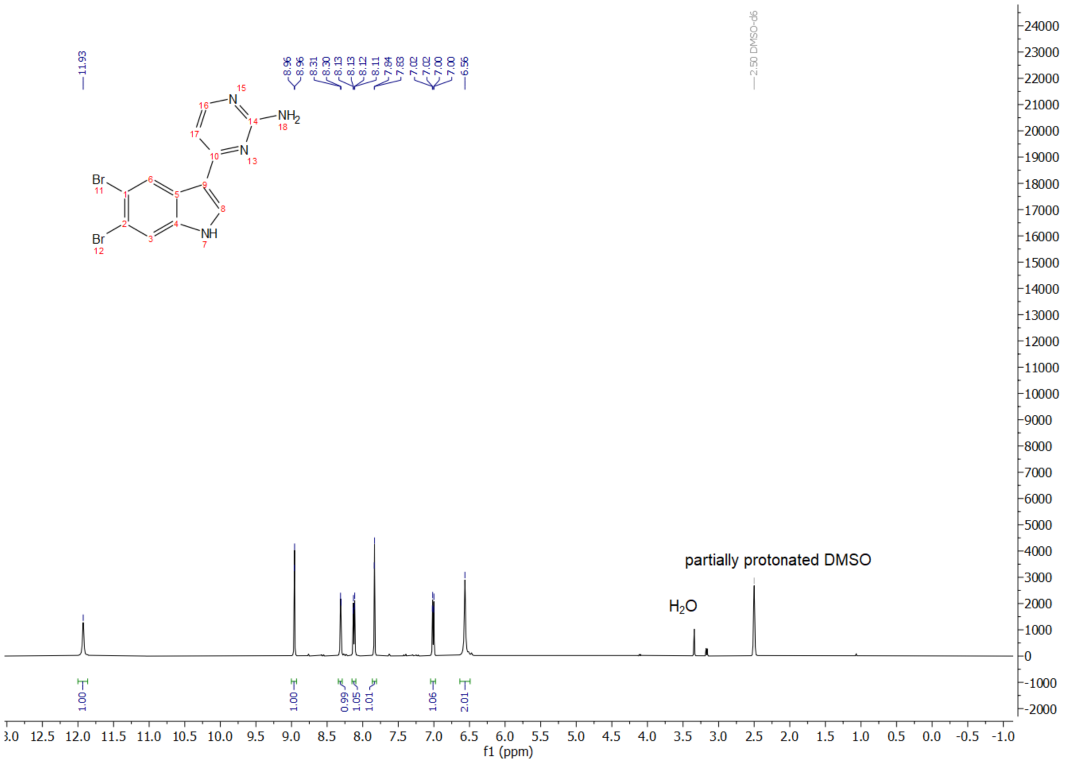

^{13}C NMR-spectrum of 4-(5,6-dibromo-1*H*-indol-3-yl)pyrimidin-2-amine (meridianin F) (**3f**) (75 MHz, DMSO-d_6, 293 K).

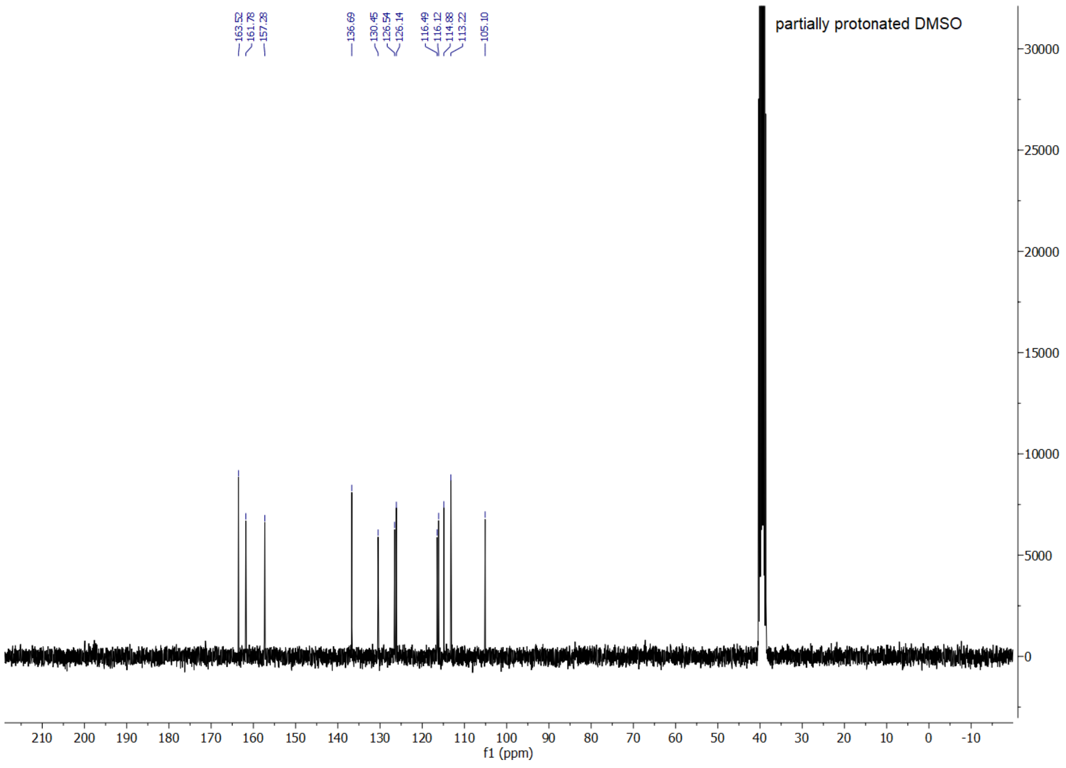

^{1}H NMR-spectrum of 4-(1*H*-indol-3-yl)pyrimidin-2-amine (meridianin G) (**3g**) (600 MHz, DMSO-d$_6$, 293 K).

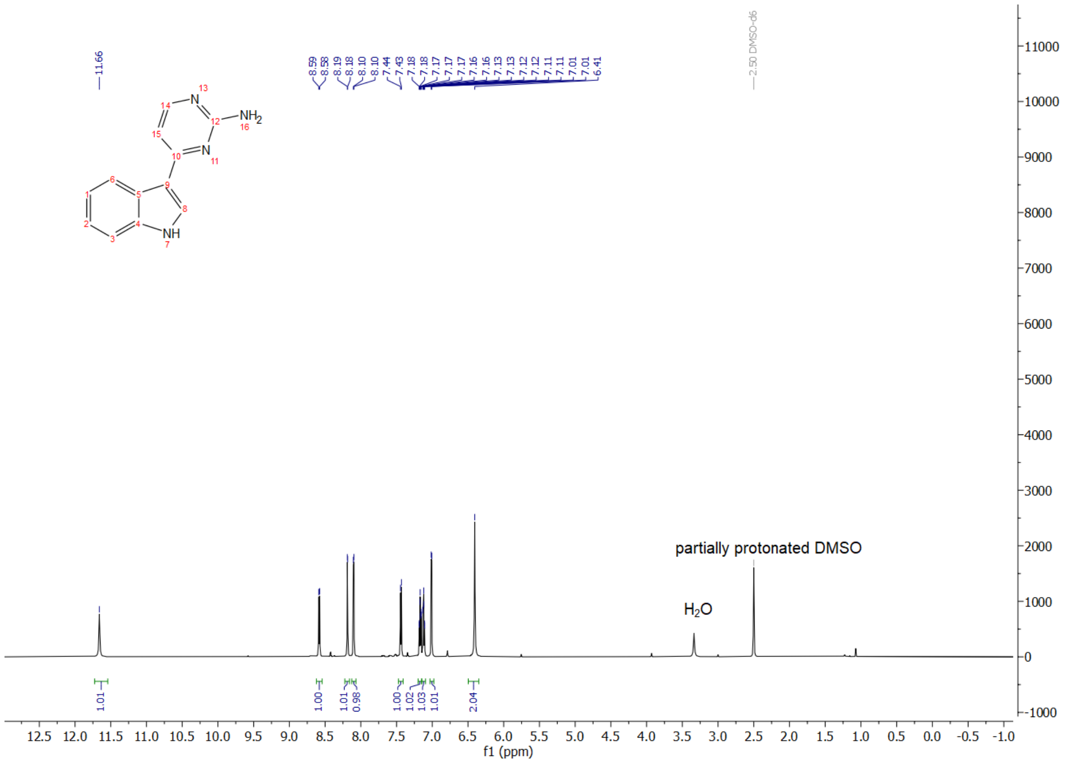

^{13}C NMR-spectrum of 4-(1*H*-indol-3-yl)pyrimidin-2-amine (meridianin G) (**3g**) (150 MHz, DMSO-d$_6$, 293 K).

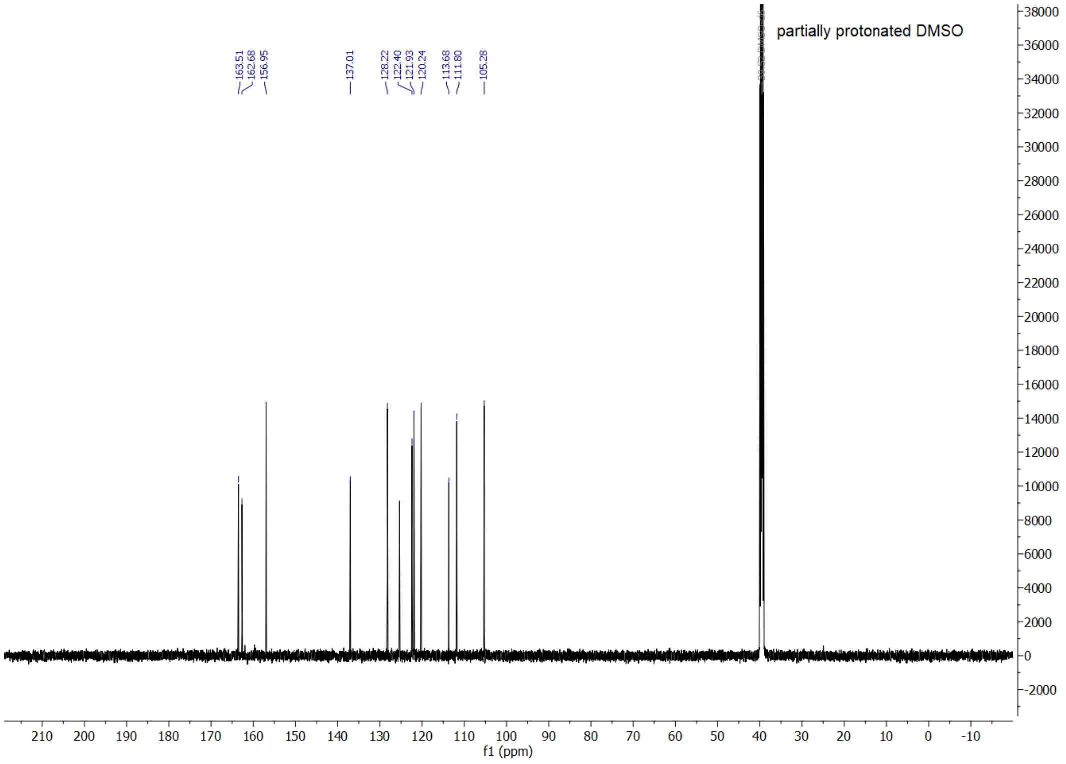

^{1}H NMR-spectrum of 3,5-bis(5-methoxy-1*H*-indol-3-yl)pyridine (**5**) (300 MHz, DMSO-d$_6$, 293 K).

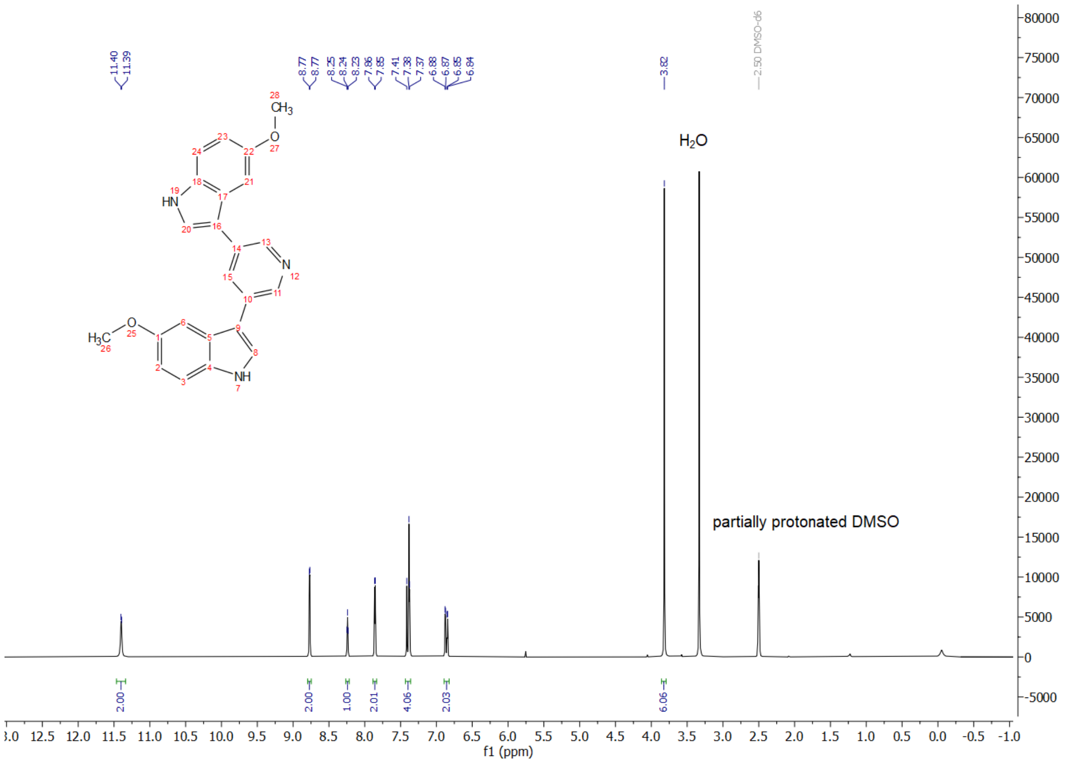

^{13}C NMR-spectrum of 3,5-bis(5-methoxy-1*H*-indol-3-yl)pyridine (**5**) (75 MHz, DMSO-d$_6$, 293 K).

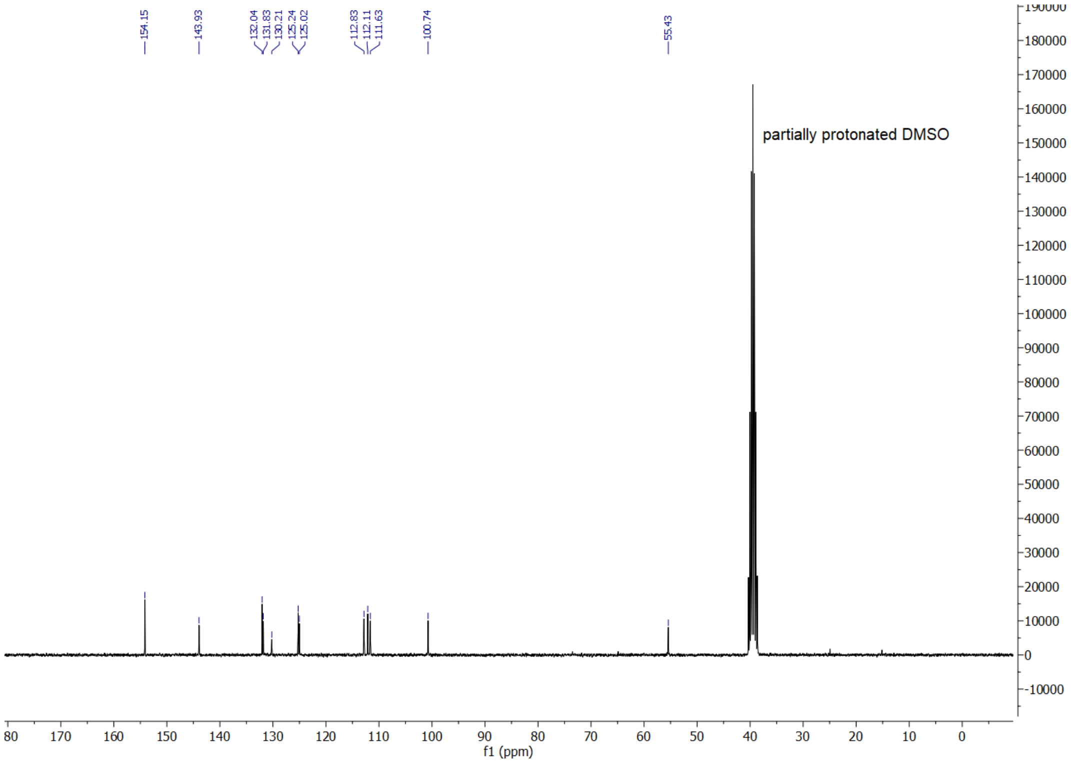

^{1}H NMR-spectrum of 3,3'-(pyridine-3,5-diyl)bis(1*H*-indol-5-ol) (scalaridine A) (**6**) (300 MHz, DMSO-d_6, 293 K).

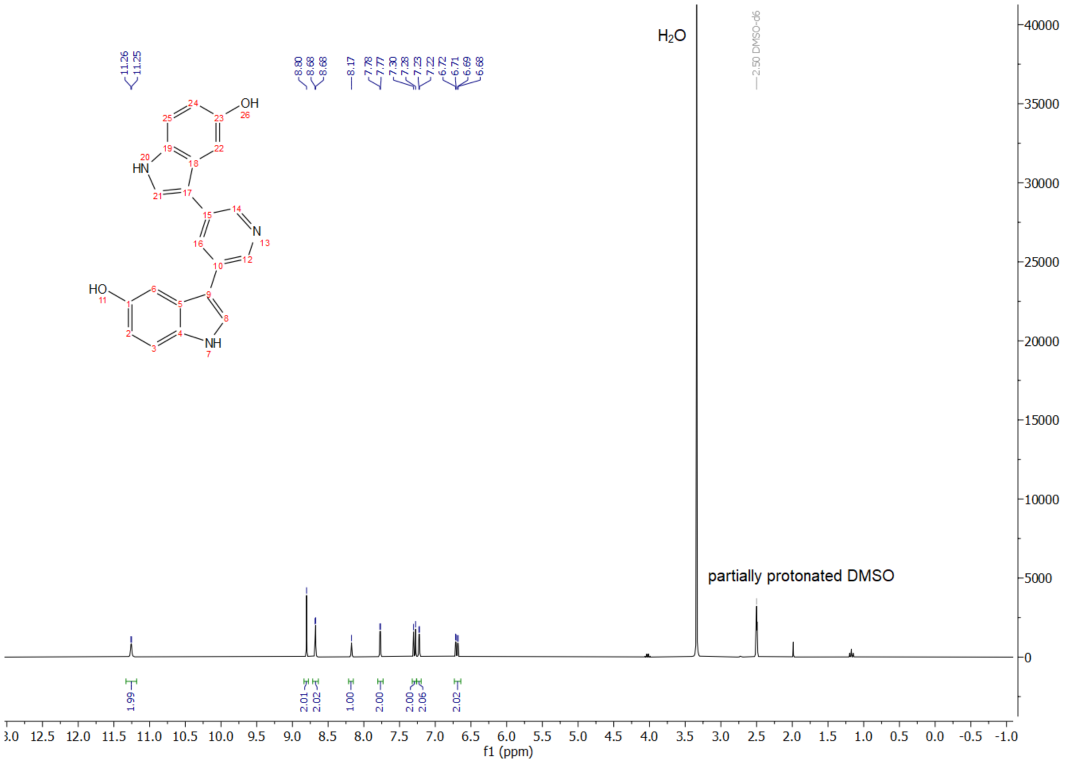

^{13}C NMR-spectrum of 3,3'-(pyridine-3,5-diyl)bis(1*H*-indol-5-ol) (scalaridine A) (**6**) (75 MHz, DMSO-d_6, 293 K).

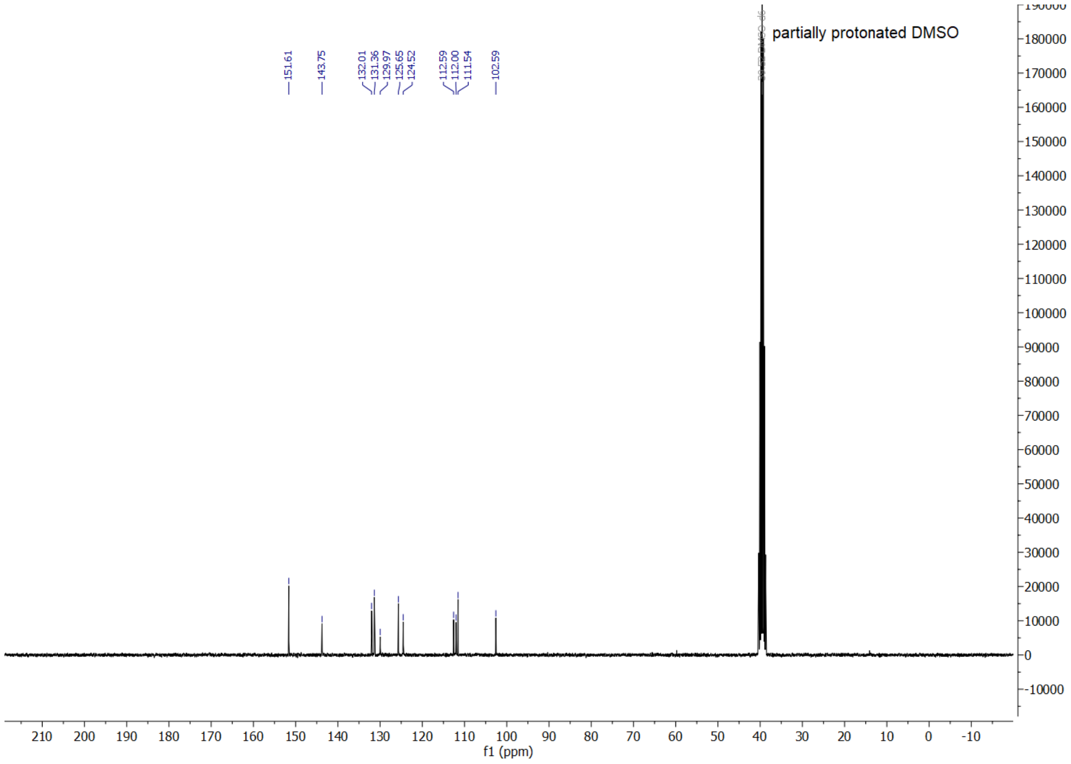

S5 References

[1] Witulski, B.; Buschmann, N.; Bergsträßer, U. *Hydroboration and Suzuki–Miyaura Coupling Reactions with the Electronically Modulated Variant of an Ynamine: The Synthesis of (E)-β-Arylenamides*, *Tetrahedron* **2000**, *56*, 8473-8480. DOI: https://doi.org/10.1016/S0040-4020(00)00773-0.

[2] Tasch, B.O.A.; Merkul, E.; Müller, T.J.J. *One-Pot Synthesis of Diazine-Bridged Bisindoles and Concise Synthesis of the Marine Alkaloid Hyrtinadine A*, *Eur. J. Org. Chem.* **2011**, *2011*, 4532-4535. DOI: https://doi.org/10.1002/ejoc.201100680.

[3] Tasch, B.O.A.; Antovic, D.; Merkul, E.; Müller, T.J.J. *One-Pot Synthesis of Camalexins and 3,3'-Biindoles by the Masuda Borylation–Suzuki Arylation (MBSA) Sequence*, *Eur. J. Org. Chem.* **2013**, *2013*, 4564-4569. DOI: https://doi.org/10.1002/ejoc.201300133.

[4] Hilbert, G.E.; Johnson, T.B. Research on pyrimidines. CXIII. An improved method for the synthesis of cytosine 1. *J. Am. Chem. Soc.* **1930**, *52*, 1152-1157. DOI: 10.1021/ja01366a051.

[5] Kofler, A.; Kolšek, J. *Beitrag zur mikroskopischen Identifizierung organischer Stoffe nach L. Kofler. IV*, *Microchim. Acta* **1970**, *58*, 1063-1088. DOI: https://doi.org/10.1007/BF01225745.

[6] Fresneda, P.M.; Molina, P.; Bleda, J.A. Synthesis of the indole alkaloids meridianins from the tunicate Aplidium meridianum. *Tetrahedron* **2001**, *57*, 2355-2363. DOI: https://doi.org/10.1016/S0040-4020(01)00102-8.

[7] Karpov, A.S.; Merkul, E.; Rominger, F.; Müller, T.J.J. Concise Syntheses of Meridianins by Carbonylative Alkynylation and a Four-Component Pyrimidine Synthesis. *Angew. Chem. Int. Ed.* **2005**, *44*, 6951-6956. DOI: https://doi.org/10.1002/anie.200501703.

[8] Sperry, J. A concise synthesis of meridianin F. *Tetrahedron Lett.* **2011**, *52*, 4537-4538. DOI: https://doi.org/10.1016/j.tetlet.2011.06.073.

[9] Merkul, E.; Schäfer, E.; Müller, T.J.J. Rapid synthesis of bis(hetero)aryls by one-pot Masuda borylation-Suzuki coupling sequence and its application to concise total syntheses of meridianins A and G. *Org. Biomol. Chem.* **2011**, *9*, 3139-3141. DOI: https://doi.org/10.1039/c1ob05310h.

[10] Kim, S.H.; Sperry, J. Synthesis of scalaridine A. *Tetrahedron Lett.* **2015**, *56*, 5914-5915. DOI: https://doi.org/10.1016/j.tetlet.2015.09.033.
